# The behavior of adult *Drosophila* in the wild

**DOI:** 10.1371/journal.pone.0209917

**Published:** 2018-12-31

**Authors:** Luis Soto-Yéber, José Soto-Ortiz, Pablo Godoy, Raúl Godoy-Herrera

**Affiliations:** 1 Departamento de Ciencias Básicas, Facultad de Ciencias, Universidad del Bío-Bío, Sede Chillán, Campus Fernando May, Avenida Coihueco S/N, Chillán, Chile; 2 Programa de Genética Humana, ICBM, Facultad de Medicina, Universidad de Chile, Independencia 1027, Santiago, Chile; University of California Santa Barbara, UNITED STATES

## Abstract

Little is known about how *Drosophila* adults behave in the wild, including mating, allocation of food and space, and escape from predators. This lack of information has negative implications for our ability to understand the capabilities of the nervous system to integrate sensory cues necessary for the adaptation of organisms in natural conditions. We characterized a set of behavioral routines of *D*. *melanogaster* and *D*. *simulans* adults in three ecologically different orchards: grape, apple and prickly pear. We also investigated how the flies identify conspecifics and aliens in the wild to better understand relationships between group formation and adaptation of *Drosophila* to breeding sites. We characterized the locations by recording in each orchard humidity, temperature, illumination conditions, pH of fruits, the presence/absence of other *Drosophila* species and the predator ant *Linepithema humile*. Our findings suggest that the home range of these species of *Drosophila* includes decaying fruits and, principally, a variety of microhabitats that surround the fruits. The ecological heterogeneity of the orchards and odors emitted by adult *D*. *melanogaster* and *D*. *simulans* influence perch preferences, cluster formation, court and mating, egg-laying site selection, and use of space. This is one of the first large examinations of the association between changing, complex environments and a set of adult behaviors of *Drosophila*. Therefore, our results have implications for understanding the genetic differentiation and evolution of populations of species in the genus *Drosophila*.

## Introduction

Interactions between parts of an organism and between complete organisms intersect in the concept of the biological organization, extending from the molecular level, such as assemblages of chemosensory proteins in *Vibrio cholerae*, to grouped distributions of animals in the wild [1, 2]. Such reciprocal influences ensure that biological organization is expressed in a coordinate manner in relation to environmental changes [3]. How animals act to affect survival, distribution and behavior of other animals is a central problem in ecology and evolution [4–7]. Association with conspecifics provides an individual animal a variety of benefits, such as opportunities to mate, the construction of habitats such as nests, and protection from predators. Conspecific aggregations may lead, however, to competition for food and space that affects individual fitness. In addition to a variety of signals emitted and processed by animals to attract and evade other animals, the heterogeneous distribution of resources may favor interactions between individuals [8]. The ability to move is another factor involved in interactions between animals [9–11]. Therefore, the capability to attract conspecifics and avoid or evade adults of other species has a principal role in the adaptation of animal species in natural conditions and has implications ranging from formation of social groups to the emergence of new species.

In this context, it is surprising that little research has been reported on how *Drosophila* adults behave in the wild. For example, associations with conspecifics and aliens have rarely been studied in wild populations of *Drosophila* by observation and experiment. Such an omission necessarily hinders and weakens comprehension of the role of behavior in the evolution of species in the genus *Drosophila* [12]. Larvae and imagoes have a remarkable variety of receptors and brain structures that process miscellaneous stimuli [13], suggesting an inherent flexibility to adapt to a wide range of complex environmental situations. In fact, responses to the odors emitted by *Drosophila* larvae are critical for the identification of conspecifics and aliens [14–16]. Species-specific odorants emitted by larvae of *D*. *melanogaster*, *Drosophila simulans*, *Drosophila buzzatii*, *Drosophila hydei* and *Drosophila pavani* participate in conspecific aggregation and provide cues regarding food availability and the physical features of pupation sites [17–19]. In larvae of *D*. *melanogaster*, *Orco*, *Syn*^*97CS*^ and *rut* loci are involved in cluster formation, orienting navigation toward conspecifics [15]. In this same species, a single larval chemosensory neuron is critical for detecting long-chain fatty acids that attract conspecifics [20]. Natural populations of *D*. *melanogaster* show genetic variation in their ability to identify conspecific larvae, suggesting that this behavior is under evolutionary selection [15,19]. Laboratory studies on the neurobiology of courtship and mating, egg-laying site selection and foraging confirm the importance of odor cues in the behavior of *Drosophila* [14–16, 21–23]. Adults of a number of *Drosophila* species are attracted by yeast odors, leading to aggregation around food in the wild [24]. These findings support the hypothesis that odor-based behavior of larvae and adults is important in the ecology and evolution of *Drosophila* and contribute to adaptation to the variable and changing environments in which these two life-stages live.

We detected groups of *D*. *melanogaster* adults away from clusters composed of *D*. *simulans* flies in a grape orchard, suggesting that imagoes of the two species can identify conspecifics and aliens at distance. Recognition could be based on species-specific odorants [14, 25, 26]. For adult *D*. *melanogaster* and *D*. *simulans* that coexist in the same orchard, the referenced clusters could favor conspecific mating and prevent hybridization between species [27]. This notion requires that, for example, species-specific odorants attract conspecifics, repelling flies of alien species. We investigated this proposition by observing and comparing routines of *D*. *melanogaster* and *D*. *simulans* adults in three ecologically different orchards. We also taxonomically classified groups of flies collected in the orchards. We substantiated our observations by testing the capability of virgin and non-virgin *D*. *melanogaster* and *D*. *simulans* adults to recognize at distance other individuals. We discuss some consequences of such behavior for the ecology and evolution of species in the genus *Drosophila*.

## Materials and methods

### Orchards

**Fig 1:**
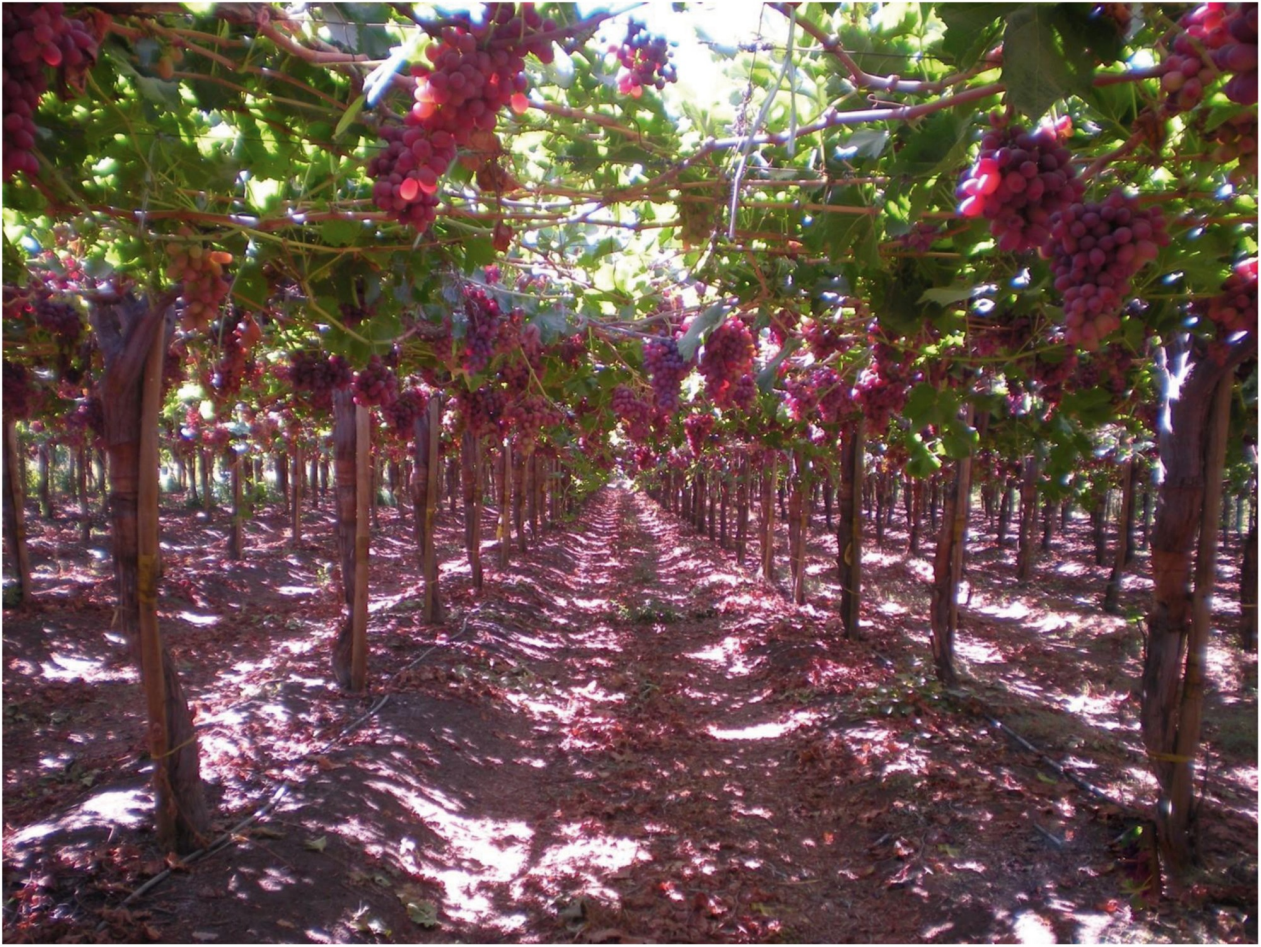
A grape orchard (*Vitis vinifera*) located in Til-Til. The plants are Sultanina variety.

**Fig 2:**
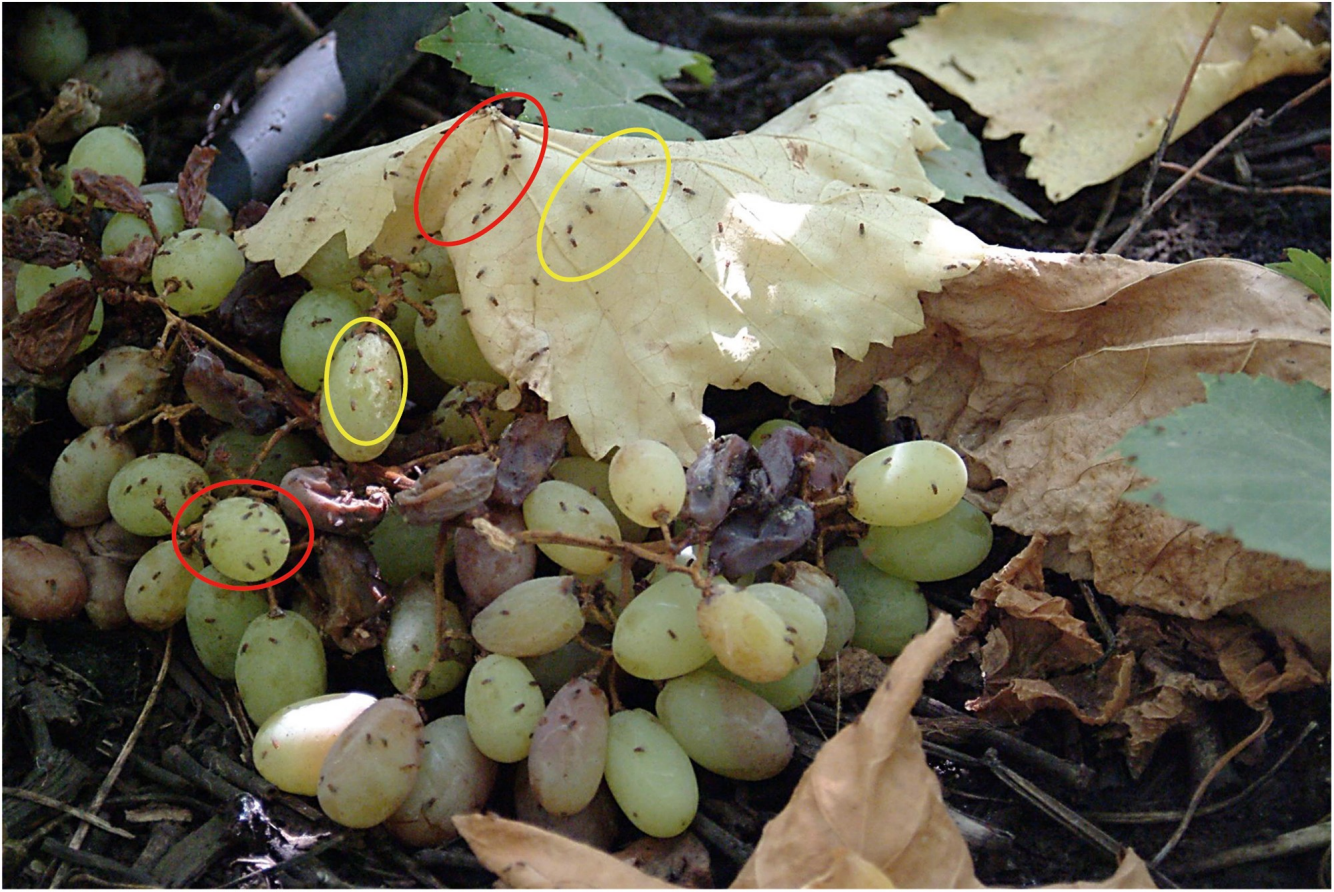
Non-random grouped distribution of adults of *D*. *melanogaster* and *D*. *simulans* on dry leaves and grape grains fallen on the ground. The orchard is in Fig 1. Adult flies of *D*. *melanogaster* are enclosed by a red oval; those of *D*. *simulans* are encircled by a yellow oval.

**Fig 3:**
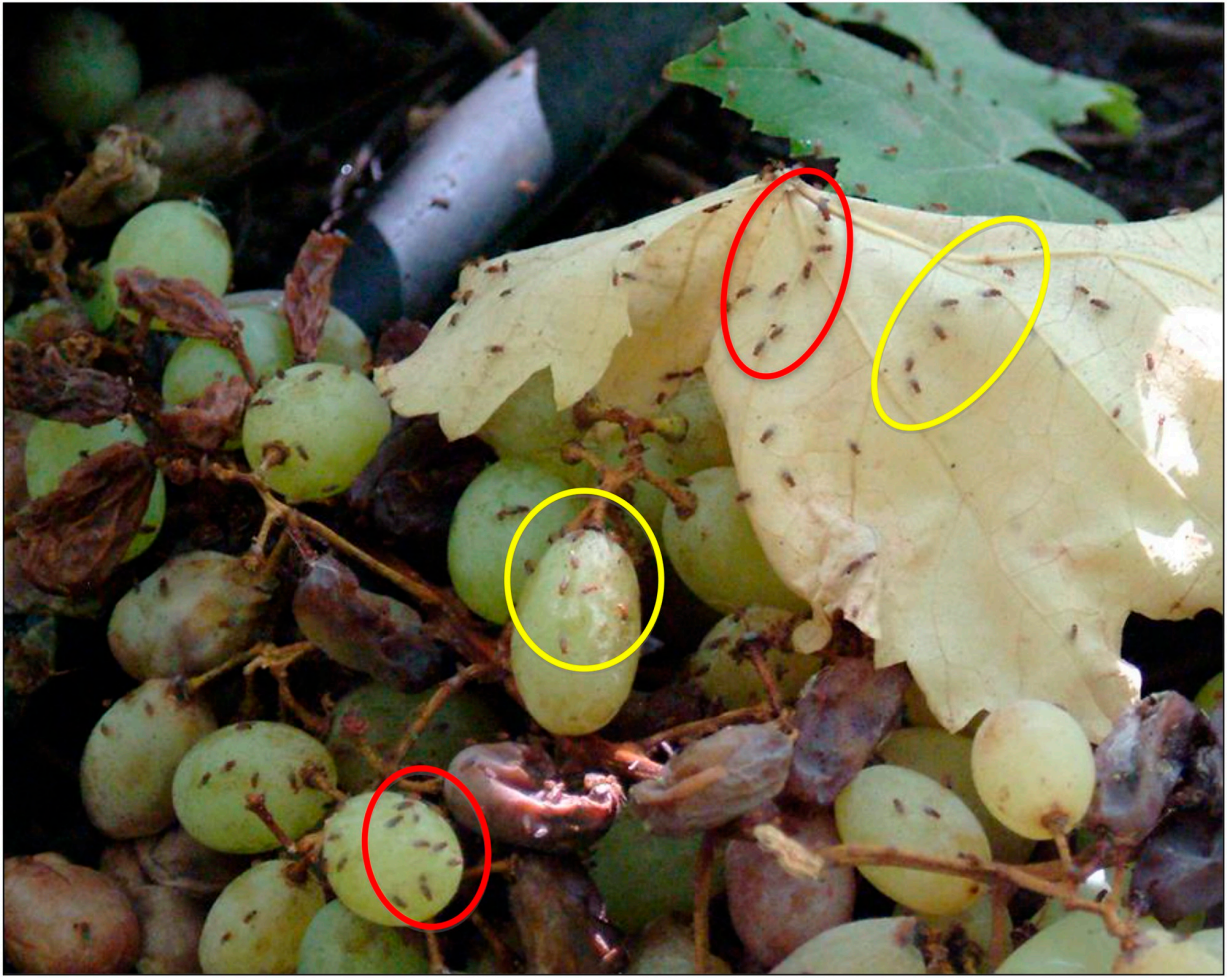
The same photograph of Fig 2 at double magnification.

**Fig 4:**
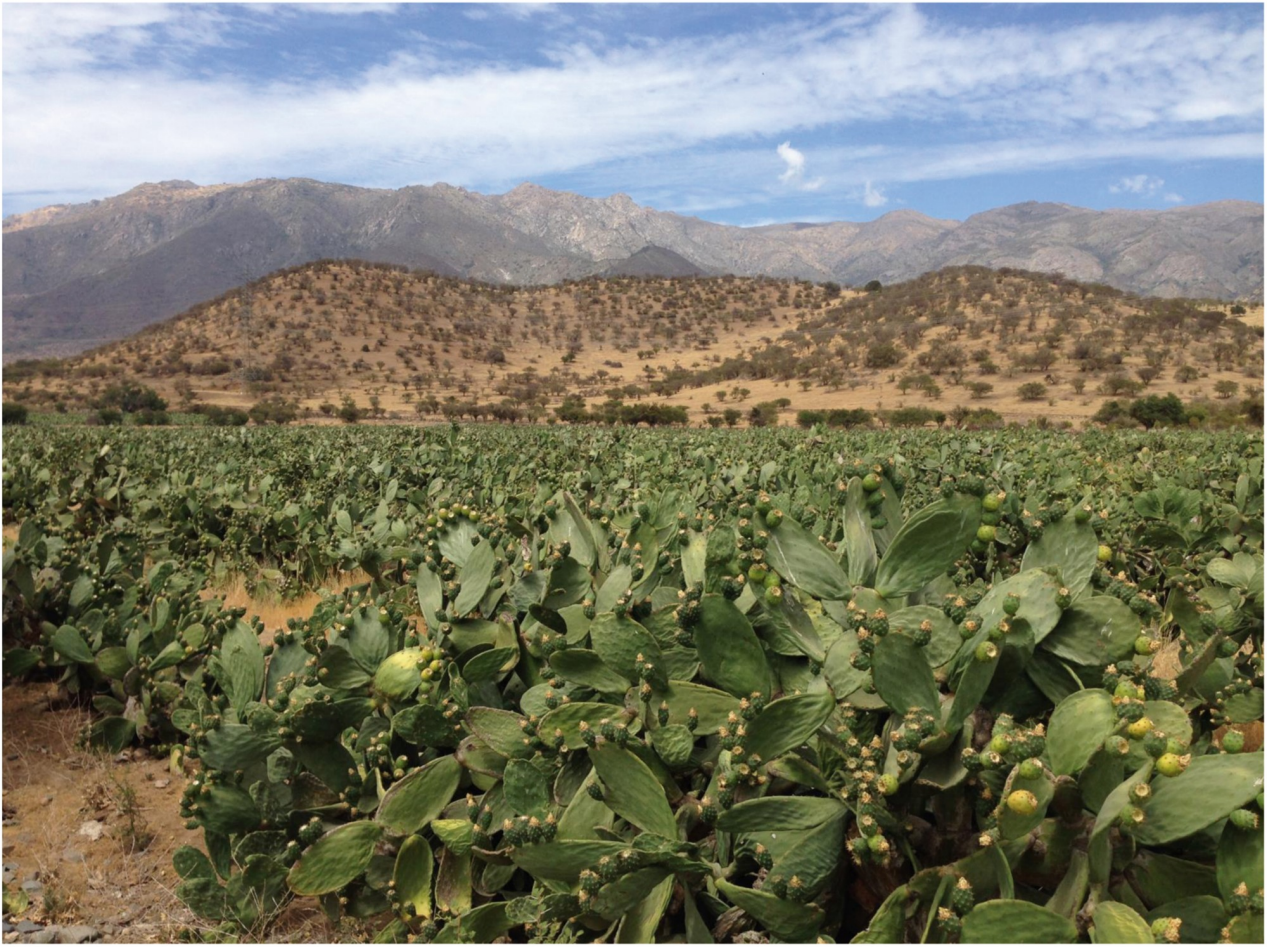
The prickly pear orchard (*Opuntia ficus-indica*) examined in this study. The orchard is located in Til-Til, 2 km away from the grape orchard shown in Fig 1.

We observed routines of *Drosophila* adults in the wild in Til-Til, 33° 05’ 00” S 50 km Northwest Santiago at the end of the Chilean summer and in autumn (March to May). In these months, *Drosophila* populations in Chile reach their peak abundance [28]. Observations were made between 9:00 am and 6:00 pm in three types of orchards: (i) grape (*Vitis vinifera*, Sultanina, Red Globe and Crimson Seedless varieties; Figs 1–3), (ii) apple (*Malus domestica*, Red Delicious variety) and (iii) prickly pear (*Opuntia ficus-indica*, Fig 4). We are grateful to Don Juan Ignacio Herrera and Don Gonzalo Herrera, owners of the Fundo La Capilla in Til-Til, who gave permission to collect flies and fruits, for their tolerance while we conducted our study on their land.

We recorded humidity and temperature at different sites in each of the orchards (N = 16 measures per orchard and parameter). The pH of mature grape, apple, prickly pear fruits hanging from the plants and fresh pieces of cladodes 5 cm in diameter cut from the plant (N = 50 units per type of fruit and tissue) was also recorded. We followed the same procedure for decaying fruits and tissues.

### Fly clusters in the wild

*Drosophila* adults were grouped in the grape orchard. Lying on the ground, we crawled toward clusters of flies perched on decaying grape bunches and the dry leaves of grape plants (Figs 2 and 3) to prevent the flies from detecting our presence and fleeing. By using a 30 x 0.5 cm (length x width) glass aspirator, we carefully collected each group of flies [29]. This procedure was also employed to collect flies in the apple orchard. Some flies escaped after detecting our presence and so were not collected from those groups; escaped adults may have belonged to other *Drosophila* species. In the prickly pear orchard, an abundance of cladode thorns were a formidable obstacle that impeded use of the aspirator to collect adults. However, with great patience, we managed to collect some flies. Each cluster was deposited into a 10 x 2.5 cm (length x width) labeled vial. Each vial contained 2.5 cm^3^ of *Drosophila* Burdick’s medium [30].

By examining external genitalia and morphological traits, we taxonomically classified the flies deposited in each vial [31]. The food was also scanned to search for eggs and larvae. A Leica M7 stereomicroscope was used for these purposes. Vials with pre-adults were maintained at 22 °C until emergence, and the imagoes were taxonomically identified. The possibility that some emerged adults could be interspecific hybrids was also scrutinized [32, 33]. The flies were individually transferred to a vial filled with *Drosophila* medium. When a new generation did not emerge, the parents were classified as interspecific hybrids. We did not determine whether the collected flies were virgins.

### Observations in the wild

We directed our attention to the behavior of adults in grape, apple and prickly pear orchards between 9:00 am and 6:00 pm (Figs 1–4). In the grape and apple orchards, most flies exhibited very little movement. By contrast, *Drosophila* adults in prickly pear orchards were mobile, moving from place to place. In the grape and apple orchards, we observed (i) courtship and mating behavior and (ii) a careful scan of the fruits (a description of the behavior is provided in the Results section). Fermented areas of the fruit were explored approximately twice as long as non-fermented areas. We examined such fruits under a stereomicroscope to locate eggs and larvae (see Results). In the prickly pear orchard, we observed courtship and feeding. We also observed that males and females met on grape, apple, and prickly pear fruits in which the decay process had started. We presumed that the flies were feeding. We carefully collected the groups and identified sex and species. We also looked for aggressive encounters between flies [34]. The *Drosophila* adults that we observed seemed vigorous, agile and attentive to the ecological quality of breeding sites.

### Stocks

We formed strains of *D*. *melanogaster* and *D*. *simulans* (subgenus *Sophophora*, *melanogaster* Group) with flies collected in the grape orchard (see above). Each stock was formed with 3 males and 3 females of *D*. *melanogaster* and *D*. *simulans* aspirated at random from each of two vials containing flies collected from grapes of the Sultanina variety (N = 12 adults; Figs 2 and 3). A similar number of males and females were taken from vials containing flies collected from dry grape leaves. Thus, each stock included 24 individuals. Before being introduced into culture bottles, adults were again taxonomically checked. The amount of genetic variability in the stocks was not estimated. The flies used in the experiments reported below were the third or fourth generation of the cultures established in the laboratory.

### Experiments

**Fig 5:**
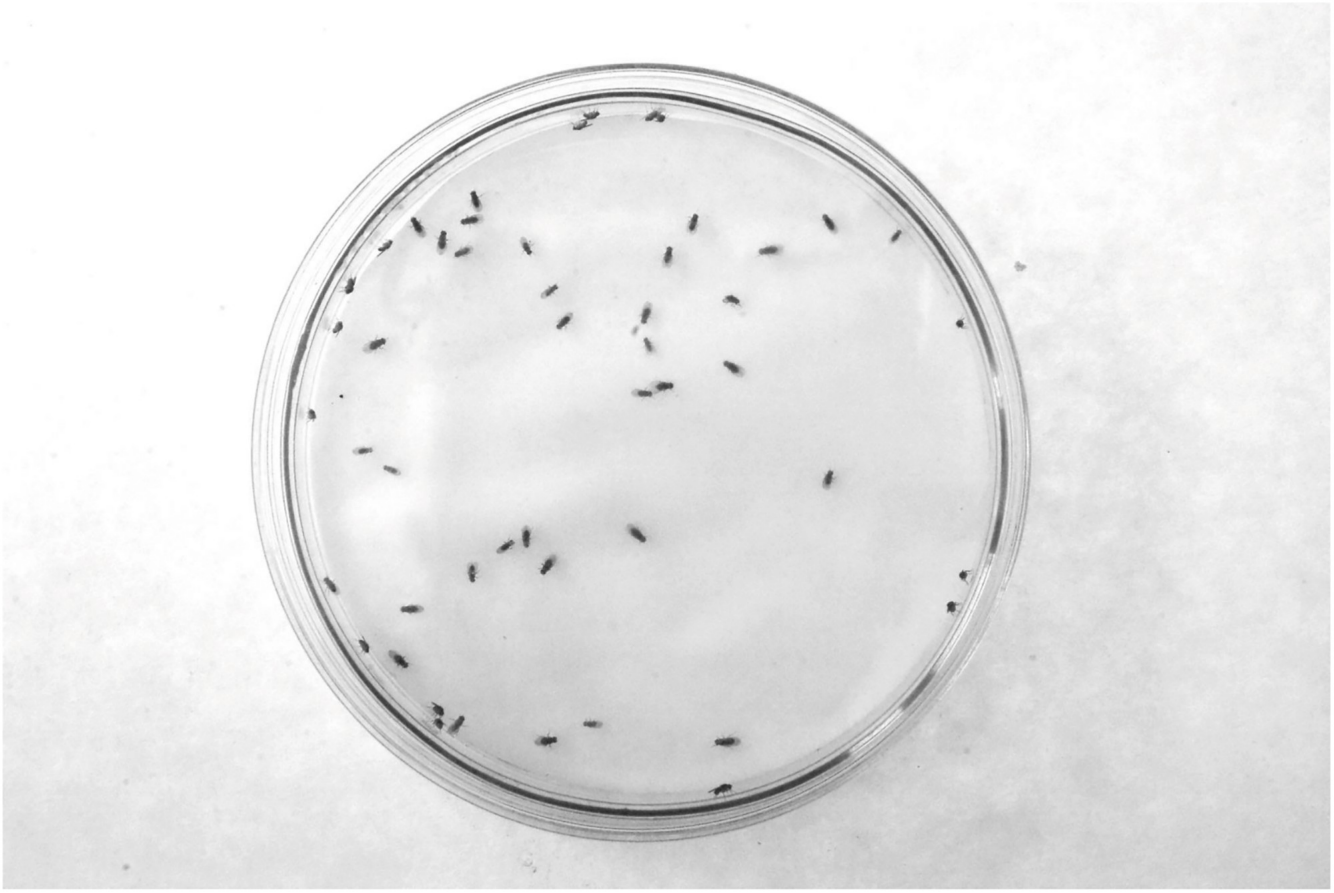
Adult *D*. *melanogaster* grouped in a Petri dish. The adults (N = 50 males) shown belong a strain formed with individuals collected in the grape orchard (Fig 1).

To determine whether *D*. *melanogaster* and *D*. *simulans* associated in the laboratory similar to those detected in the wild, we introduced 8, 10, 25 and 50 non-virgin males and females of 4–5 day old individuals of each species into 10-cm-diameter glass Petri dishes. Adults of both sexes of both species formed distinct clusters distributed within the Petri dishes (Fig 5). Individual perch preferences in the Petri dishes of virgin and non-virgin 4–5 day old flies of these stocks were determined (N = 50 individuals per sex and species). For dispersal, groups of 10 individuals were used in 10 independent replicates (details below). Virgin flies were collected without anesthesia 3–4 h after emergence from pupae. They were aged, the two sexes separately, until they were 4–5 days of age in vials filled with synthetic *Drosophila* food [30]. Non-virgin males and females were also separated without anesthesia 2 days after emergence and aged in vials with food until 4–5 days old. Experiments on perch preferences and dispersal were carried out at 20 °C under homogenous lighting conditions and 80% humidity. Illumination conditions were monitored with a Leitz-Westlar Microsix-L 2875 photometer.

### Perch preferences

Each Petri dish offered the flies three different places to perch: (i) the lid of the plate; (ii) a vertical surface, namely, the wall of the Petri dish; and (iii) the bottom of the plate. Each fly tested was aspirated into the Petri dish through a 0.5 cm diameter hole made in the wall of the Petri dish. The hole was stoppered with a cotton wool plug to prevent flies escaping from the dish. Ten minutes later, once flies had recovered from handling and had acclimated to the Petri dish, we recorded the location (ceiling, wall, floor) where each individual perched. For each fly tested, a new Petri dish was used.

The controls for perch preferences of virgin and non-virgin *D*. *melanogaster* and *D*. *simulans* adults consisted of Petri dishes without congeners and alien fly odors (treatment 1). In treatments 2 and 3, Petri dishes contained, respectively, 8 non-virgin or 8 virgin *D*. *melanogaster* males for 30 min. After this time, the flies were liberated; eight was the mean number of adults composing clusters observed in the wild (see Results). In treatments 4 and 5, the Petri dishes contained, respectively, non-virgin and virgin conspecific female odors. Treatments 2–5 were repeated with 8 non-virgin and virgin individuals each of *D*. *simulans* introduced into the Petri dishes for 30 min (treatments 6–9). The experiment was repeated with *D*. *melanogaster* females. The whole experiment was also run for both sexes of *D*. *simulans*.

### Dispersal orientation

**Fig 6:**
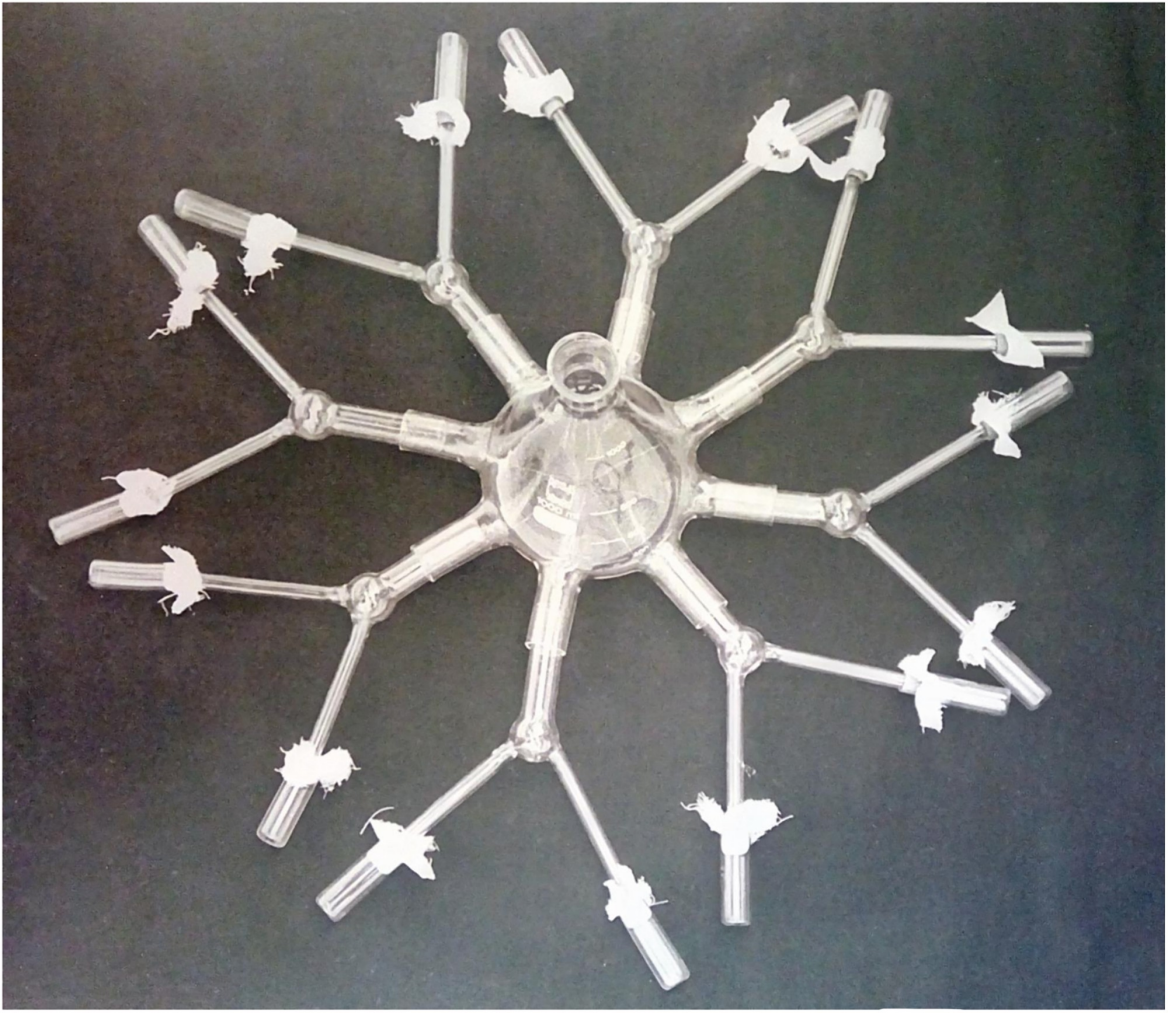
Apparatus used to study orientation of dispersal movements in adults of *D*. *melanogaster* and *D*. *simulans*. The flies were confronted with odors of the two species. The distal end of each Y-tube was connected to an empty cell impregnated with odors of the species.

We also studied the orientation of the movements of the sexes of *D*. *melanogaster* and *D*. *simulans* stimulated at distance by odors of conspecifics and aliens. The apparatus used is depicted in Fig 6. It consisted of a 1-liter conical flask fitted at the base with 8 regularly spaced tubes. An 18-cm-Y-shaped long tube was connected to each tube. The distal end of each Y-tube was fitted with a cell. In treatment 1, 10 non-virgin (or virgin) *D*. *melanogaster* males (or females) were deposited into the flask when the cells were empty and had no odors (first control treatment). The number of flies in each of the Y-tubes was recorded 10 min later. In treatment 2, each empty cell had previously contained 8 virgin (or non-virgin) males (or females) of the same species on one side for 30 min, and the corresponding cells on the opposite side had contained 8 virgin (or non-virgin) *D*. *simulans* males (or females) for a similar time (second control treatment). In treatment 3, each empty cell on one side had 8 virgin (or non-virgin) conspecific males (or females), and the corresponding cells on the opposite side were empty and had no fly odors. Treatment 4 was similar to treatment 3 except that the cells on one side had contained non-virgin (or virgin) *D*. *simulans* males (or females). S1 Fig shows a diagram depicting the essays described (see also Fig 6). Each treatment was replicated 10 times. The whole experiment was repeated by testing virgin and non-virgin *D*. *simulans* males (or females). The apparatus was carefully washed and dried before each treatment and replication. The hole of the flask was plugged with a cotton wool plug to prevent flies from escaping the apparatus.

### Statistical analysis

#### Perch selection

We performed a multinomial logistic regression for the perch selection data; the dependent variable was nominal with three levels (wall, ceiling, floor). The link function was the logit function (Ln). We assumed that the log-odds followed a linear model [35]. As the base comparator, the percentage of flies detected on the floor of the Petri dishes was used. This place was the least preferred section of the dishes (see Results).

The equation was:

$$\begin{array}{l} \text{Ln}\;\left(\text{percentage}\;\text{of}\;\text{flies}\;\text{detected}\;\text{on}\;\text{the}\;\text{wall}\;\text{of}\;\text{the}\;\text{Petri}\;\text{dishes}\right)=\text{odor}\;+\;\text{sex}\;\text{+}\;\text{sexual}\\ \text{experience}\;\text{+}\;\text{species}\;\text{+}\;\text{odor}\;\text{x}\;\text{sex}\;\text{+}\;\text{odor}\;\text{x}\;\text{sexual}\;\text{experience}\;\text{+}\;\text{odor}\;\text{x}\;\text{species}\\ +\ldots \ldots \ldots \ldots ‥\text{+}\;\text{odor}\;\text{x}\;\text{sex}\;\text{x}\;\text{sexual}\;\text{experience}\;\text{x}\;\text{species} \end{array}$$

#### Box plots

**Fig 7:**
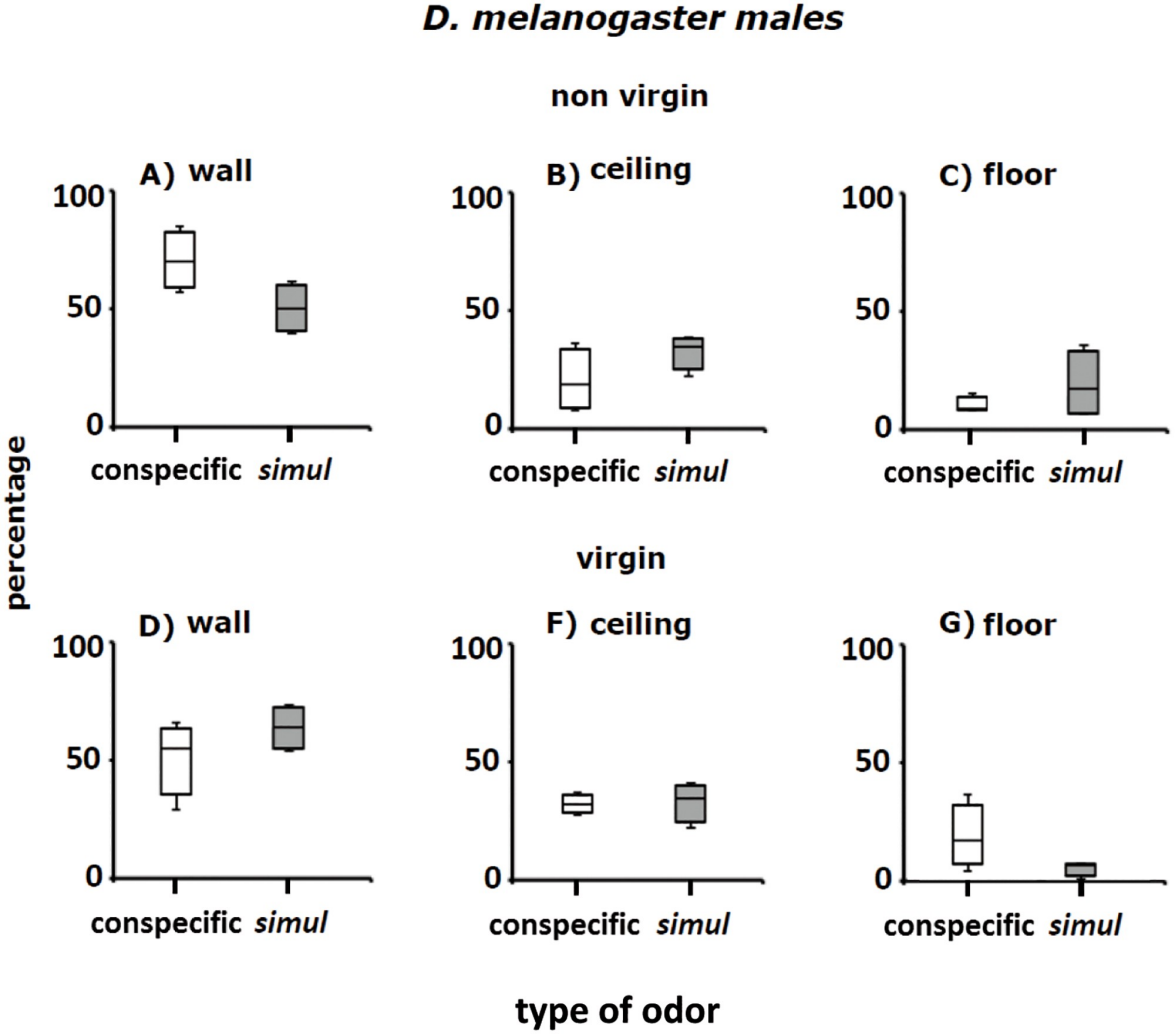
Perch preferences (percentages) of non-virgin and virgin males of *D*. *melanogaster* in Petri dishes. Perch preferences were examined in two types of Petri dishes: (i) impregnated with conspecific adult odorants (white columns), and (ii) saturated with adult odorants of *D*. *simulans* (grey columns). Fig 7 A–C and Fig 7 D–G show, respectively, percentages of non-virgin and virgin males of *D*. *melanogaster* perched on the wall, ceiling and floor of the Petri dishes. The horizontal black lines inside the boxes indicate median values. Bars outside the boxes show, respectively, maximum greatest values and minimum least values of the distributions. Conspecific is an abbreviation of Petri dishes with conspecific odors; *simul* stands for Petri dishes with odors of *D*. *simulans*.

**Fig 8:**
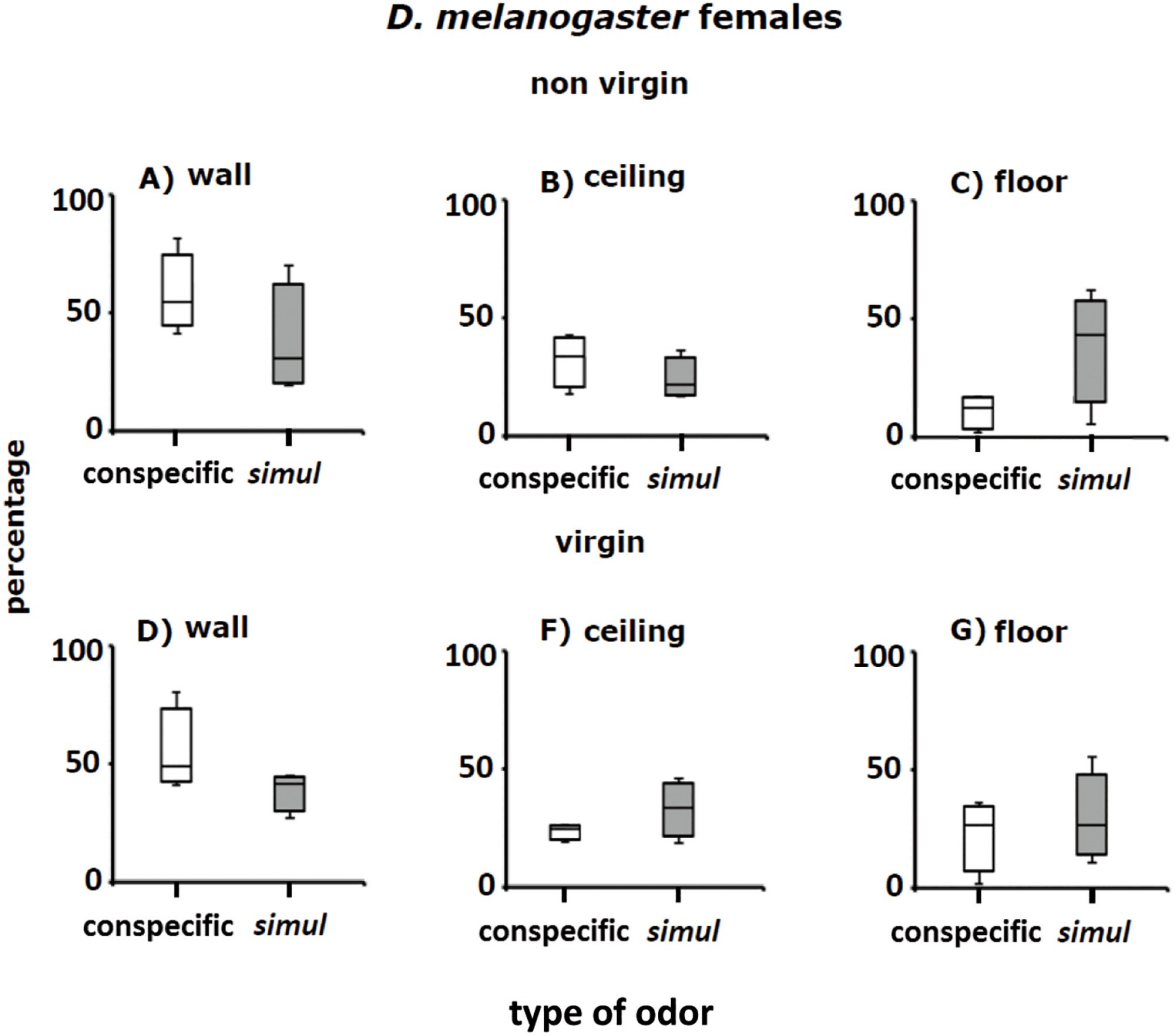
A—G. Perch preferences (percentages) of non-virgin and virgin female *D*. *melanogaster* in Petri dishes mentioned in Fig 7. White columns show distribution in Petri dishes with conspecific odors. Grey columns stand for distribution patterns in Petri dishes with odorants of adult *D*. *simulans*. See Fig 7A–7G for further details.

**Fig 9:**
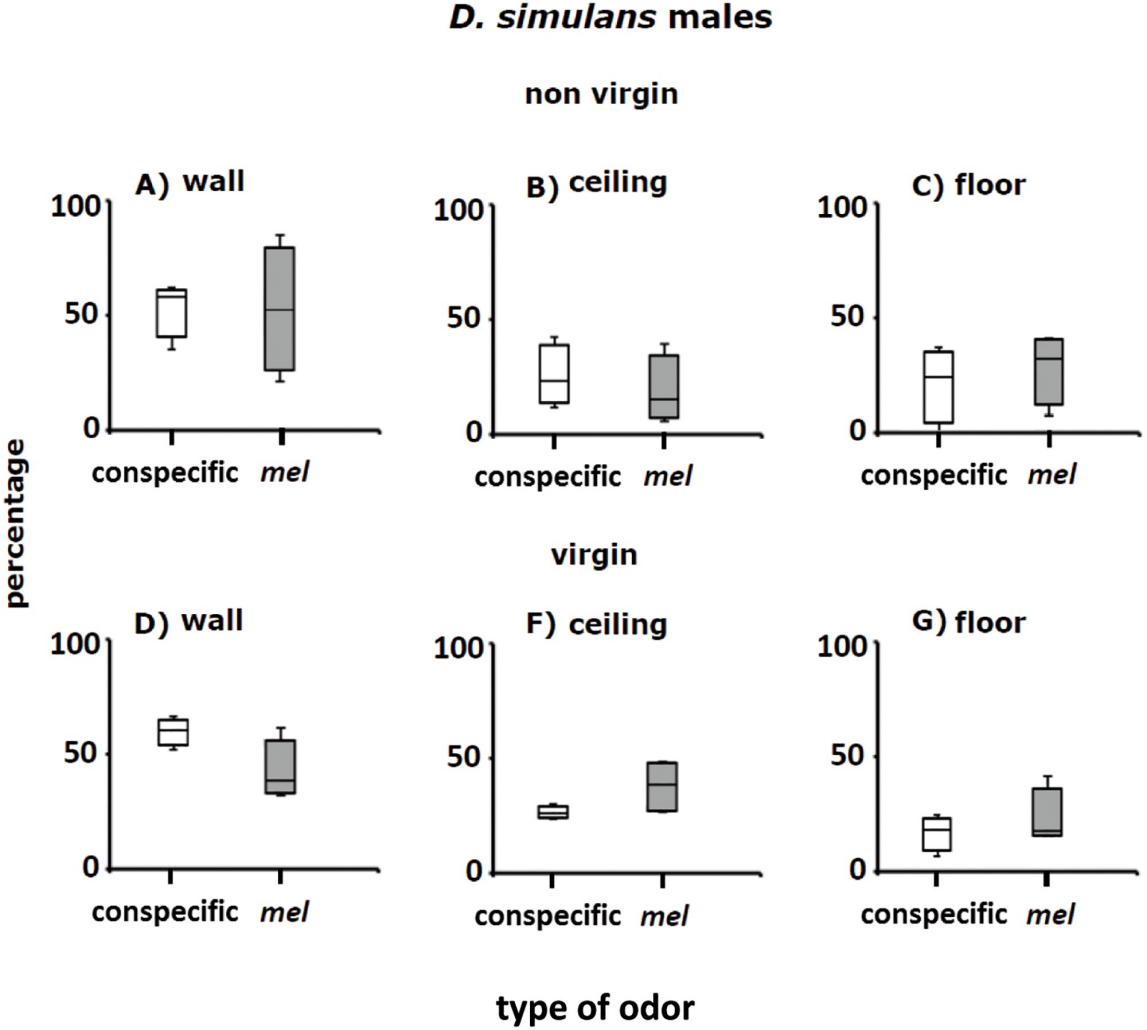
Perch preferences (percentages) of non-virgin and virgin males of *D*. *simulans* in Petri dishes with conspecific adult odorants (white columns) and adult odorants of *D*. *melanogaster* (grey columns). Conspecific alludes to Petri dishes with conspecific odors; *mel* stands for Petri dishes with adult *D*. *melanogaster* odors. See also Fig 7.

**Fig 10:**
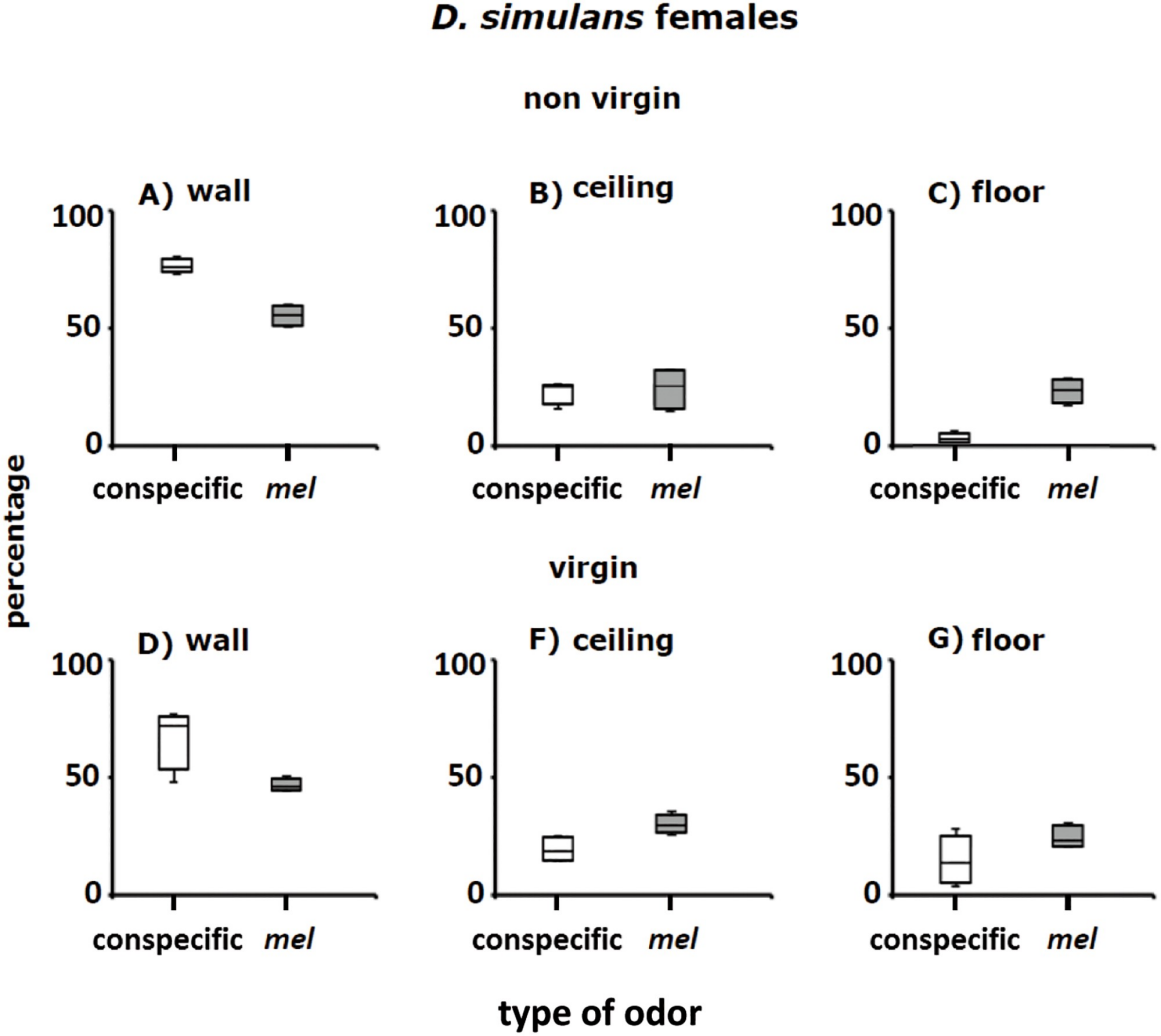
Perch preferences (percentages) of non-virgin and virgin females of *D*. *simulans* in the Petri dishes with conspecific adult odorants (white columns) and adult odorants of *D*. *melanogaster* (grey columns). See also Figs 7 and 9.

We first examined the data on perch preferences, noting that we were dealing with skewed data. We changed the scale (log scale), but the bias was not rectified. Therefore, we used the median to express the central tendency together with the lower and upper quartiles and minimum least and maximum greatest values to describe the distribution of the dataset in the applied treatments. Outlier values were not found. We built box-and-whisker plots to compare those distributions. For example, we added percentages of non-virgin males of *D*. *melanogaster* calculated from the number of flies recorded on the wall of the Petri dishes in the four treatments with conspecific odors; such a box plot was compared with the distribution of non-virgin males on the wall of the Petri dishes with *D*. *simulans* odors (Figs 7–10).

#### Statistical analysis of dispersal

Dispersal data were analyzed by applying a generalized linear model based on a binomial distribution because the dependent variables are binary: (i) number of flies in the flask and (ii) number of flies in the Y-tubes. For each variable, we conducted an analysis based on the linear independent explanatory variables of odor, sex, sexual experience, species, and the corresponding interactions. The link function was the logit function for binary data [35]. The equation was:

$$\begin{array}{l} \text{Ln}\;\left(\text{number}\;\text{of}\;\text{flies}\;\text{within}\;\text{the}\;\text{flask}\right)=\text{odor}\;\text{+sex}\;\text{+}\;\text{sexual}\;\text{experience}\;\text{+}\;\text{species}\;\text{+}\;\text{odor}\;\text{x}\\ \text{sex}\;\text{+}\;\text{odor}\;\text{x}\;\text{sexual}\;\text{experience}\;+\;\text{odor}\;\text{x}\;\text{species}\;+\ldots \ldots \ldots \ldots ‥\text{+}\;\text{odor}\;\text{x}\;\text{sex}\;\text{x}\;\text{sexual}\\ \text{experience}\;\text{x}\;\text{species}. \end{array}$$

For statistical analysis we used STATA MP 14.0 for Windows 32 bits, 2015, Serial 10699393.

## Results

### Physical and chemical features of *Drosophila* breeding sites

In the grape orchard, the canopy decreases the overall impact of sunlight, but foliage density is heterogeneous, leaving sites with more or less sunlight (Fig 1). Exposure to sunlight affects the duration of fruits as breeding sites due to loss of water (Figs 2 and 3). At 2:30 pm, the mean environmental temperature in the grape orchard was 24.60 ± 2.80 °C (N = 37 measurements), environmental humidity reached 79.80 ± 9.52% (N = 48 measurements), and pH of mature, unfermented grape grains hanging from the plants was 7.0 ± 0.4 (N = 47 measurements). In totally fermented grape grains of the same variety, the pH was 2.7 ± 0.8 (52 measurements); a strong acetic acid aroma was also detected over decaying fruits. We also occasionally found adults of the predator Argentinian ant *Linepithema humile*.

In the prickly pear orchard, a canopy is absent (Fig 4). Unfermented and decaying fruit units and pieces of cladodes used as breeding sites by *Drosophila* species are all exposed to extreme solar radiation. At 2:30 pm, the mean environmental temperature was 48.20 ± 2.56 °C (34 measurements), environmental humidity reached 42.34 ± 2.07% (34 measurements), and the pH of unfermented prickly pear fruits was 6.8 ± 0.5 (52 measurements); in decaying fruits fallen on the ground, the pH was 3.4 ± 0.5 (58 measurements). The pH of unfermented cladode tissue was 7.4 ± 2.3 (48 measurements); the pH of decaying cladode tissue was 4.4 ± 1.2 (59 measurements); an acetic acid aroma was not detected. The prickly pear orchard had abundant *L*. *humile*.

In the apple orchard, the canopy casts a shadow covering an area of 2–3 m around each tree, leaving sunny places 3–5 m in diameter between neighboring trees. We found decaying apple units within and outside the shadowed area. At 2:30 pm, the mean environmental temperature in a shady habitat was 28.75 ± 2.79 °C (35 measurements), and the temperature in sunlit microhabitats was 42.56 ± 1.93 °C (31 measurements). Mean humidity reached 63.67 ± 5.87% (34 measurements), and the pH of fruit fluctuated between 6.2 ± 0.4 in non-decaying apples hanging from the trees (Red Delicious variety; 46 measurements) and 3.4 ± 1.5 in decaying fruits of the same variety (59 measurements); acetic acid aroma was not detected. No *L*. *humile* were found.

### Fly routines in the grape orchard

In the grape orchard, *D*. *melanogaster* (54.78 ± 2.87%), *D*. *simulans* (45.03 ± 6.71%) and *Drosophila busckii* (0.19 ± 0.04%) were perched on dry leaves scattered on the ground and on unfermented grains of grape forming clusters (Figs 2 and 3). The remarkable absence of movement was broken by very few attempts at courtship and copulation. A male courted a female within a cluster (Figs 2 and 3); the female left the group, walking quickly followed closely by the courtier male vibrating a wing. Other males of the same and neighboring groups joined in, aligning behind. Headed by the female, the train snaked among fly clusters. Abruptly, the courting male stopped following the female and after a few seconds, the escort train dispersed and the males returned to the groups. In other instances, the female stopped walking while the leader male increased the frequency of semicircles around the female. Males of the escort train remained near the partners, vibrating their wings. After copulation, the escort males returned to the groups. These observations suggest cooperative male courtship. Courtship and copulation were also observed on unfermented grains of grape fallen on the ground (Figs 2 and 3). However, participation of other males was not observed. We did not detect aggressive encounters between flies perched on dry leaves and on grains of grape.

On the surface of unfermented grape grains, some female *D*. *melanogaster* and *D*. *simulans* were observed scanning the fruit. The process took approximately 10.74 ± 3.51 min (45 observations). We observed that 2–3 females landed at unfermented grape grains or grains where fermentation was just starting; after 8–11 sec, one of the flies walked two or three steps and stopped, spreading the trunk and leaving the end of it in contact with the surface of the fruit. After 15–20 sec, the fly again walked two to three steps, extending the trunk until the whole of fruit surface was explored; the other flies remained motionless. We collected the scanned grains (241 units); we did not detect eggs or larvae on 206 grains (85.48%), whereas 35 grains (14.52%) had eggs or larvae. Adult *D*. *melanogaster* or *D*. *simulans* emerged from 34 of these grains (99.95%); adults of both species emerged from 1 grain. Abundant colonies of microorganisms consumed by the larvae of *Drosophila* were present on the decaying grape grains. A few large larvae were also observed moving on decaying grape grains and on the ground; presumably, they were searching for pupation sites. We also detected clusters of *Drosophila* pupae adhered to the stalks of bunches of grapes; the pupae were principally located between the grapes and the ground.

Table 1 shows the distribution of adult flies on the dry leaves of grape plants and unfermented grape grains on the ground (Figs 1–3).

**Table 1:** Number of male and female *D*. *melanogaster* and *D*. *simulans* collected from dry leaves and grains of grapes on the ground. Each collected group of flies was deposited into a vial. The sexes of individuals in each sample vial were taxonomically identified.

*Drosophila* species and type of substratum where collected										
N° Vial	dry leaves of grape plants				N° Vial	grains of grape				Total
	*melanogaster*		*simulans*			*melanogaster*		*simulans*		
	male	female	male	female		male	female	male	female	
**1**	4	5	–	–	22	2	1	–	–	12
**2**	5	3	–	–	23	1	2	–	–	11
**3**	3	2	–	–	24	3	1	_	_	9
**4**	4	3	–	–	25	–	–	2	1	10
**5**	3	4	–	–	26	–	–	2	2	11
**6**	3	5	–	–	27	2	2	–	–	12
**7**	–	–	3	4	28	–	–	3	1	11
**8**	–	–	4	4	29	–	–	4	–	12
**9**	–	–	4	3	30	–	–	1	3	11
**10**	–	–	5	4	31	–	–	–	4	13
**11**	–	–	3	4	32	3	–	–	–	10
**12**	–	–	4	4	33	2	2	–	–	12
**13**	2	4	–	–	34	–	3	–	–	9
**14**	–	–	5	3	35	–	–	–	2	10
**15**	4	1	–	–	36	3	1	–	–	9
**16**	6	3	–	–	37	–	–	1	2	12
**17**	–	–	1	6	38	3	3	–	–	13
**18**	–	–	4	4	39	2	2	–	–	12
**19**	5	1	–	–	40	1	4	–	–	11
**20**	3	3	–	–	41	2	2	–	–	10
**21**	–	7	–	–	42	1	3	–	–	11
**Total**	42	41	33	36		25	26	13	15	231

Each vial contained adult *D*. *melanogaster* or *D*. *simulans*. Hybrids between the species were not detected. These findings confirm that *D*. *melanogaster* adults distributed differentially from *D*. *simulans* in this grape orchard.

### Fly routines in the prickly pear orchard

In the prickly pear orchard (Fig 4), we found adults of *D*. *melanogaster* (13.82%), *D*. *simulans* (37.08%), *Drosophila buzzatii* (48.67%), *Drosophila nigricruria* (0.28%) and *Drosophila pavani* (0.15%). Compared to observations in the grape orchard, adults were actively moving from place to place on prickly pear fruits and cladodes. We observed courtship and mating, and feeding; fly clusters were not observed. We also detected groups of 5–6 flies of *D*. *buzzatii* on the cladodes of the plants, arranged in circles with heads directed toward the center of the circle. The functional significance of such behaviors remains to be investigated. Nearly 100% of the fallen prickly pear fruits had an orifice of 2.5 ± 0.8 cm in diameter and 1.5 ± 0.5 cm deep made by wild Chilean birds, the Austral blackbird (*Curaeus curaeus*), the Austral thrush (*Turdus falcklandii*) and the Chilean mockingbird (*Mimus thenca*). Adult and larval *Drosophila* were detected inside the holes, on the edges of the holes and on peels of the fruits.

Courtship and mating of *D*. *melanogaster* adults was observed on the peel and inside the holes of prickly pear fruits. Individuals of *D*. *simulans* were detected principally on cladodes fallen on the ground on which the decay process was starting. Cooperative male courtship as that observed in the grape orchard was not observed. We found *D*. *buzzatii* adults mating on cladodes on the plant. These results suggest that the three more abundant *Drosophila* species mate on different substrates in the prickly pear orchard.

The fruits and cladodes in the prickly pear orchard were awash with Argentinian ants (*L*. *humile*). The ants captured larval and occasionally adult *Drosophila*. We noted that, upon arrival of the ants at prickly pear fruits, *Drosophila* adults flew away. Two or three ants cooperatively captured larvae. We observed that larvae quickly detected the arrival of ants, as suggested by an increase in locomotion and a decrease of turns, followed by a rapid navigation toward the liquefied part of the fruit, where they sank and disappeared. The behavioral changes of the larvae seem to alert the adults of the ant’s arrival.

### Fly routines in the apple orchard

The dominant species in the apple orchard was *D*. *simulans* (96.10%). Adults of *D*. *melanogaster* (2.13%), *Drosophila repleta* (*repleta* species Group, 1.02%), *D*. *hydei* (*repleta* species Group, 0.56%) and a few adults of *Drosophila subobscura* (*obscura* species Group, 0.19%) were also observed. Flies were perched on decaying apples that had fallen on the ground and on herbs and dry branches distributed around such fruits. As in the grape orchard, an absence of movement was the most remarkable behavior of the adults. Clusters of flies were not detected.

### Perch site selection

#### *D*. *melanogaster* males

Fig 7A–7G shows the perch preferences of non-virgin and virgin *D*. *melanogaster* males in Petri dishes with conspecific odorants (white columns) and odorants of *D*. *simulans* (gray columns). Places available to perch include the wall, ceiling and floor of the Petri dishes as noted above. Most non-virgin and virgin *D*. *melanogaster* males perched on the wall of the Petri dishes. However, the distribution of male *D*. *melanogaster* on the wall of Petri dishes with conspecific odorants was clearly different than in the Petri dishes with *D*. *simulans* odorants, as shown by median values, lower and upper quartiles, and minimum least values and maximum greatest values of the distributions (Fig 7A and 7D. See below Statistical analysis of perch site selection and S1 Table).

In line with the above results, the distribution of non-virgin and virgin males perched on the ceiling (Fig 7B and 7F) and floor (Fig 7C and 7G) of Petri dishes with conspecific odorants (white columns) differed from the distribution in the same sites in Petri dishes impregnated with *D*. *simulans* odorants (gray columns; see below: Statistical analysis of perch site selection and S1 Table).

#### *D*.*melanogaster* females

Perch preferences of non-virgin and virgin females of *D*. *melanogaster* were also affected by odorants left by conspecific adults of the two sexes, and adult *D*. *simulans* (Fig 8A–8G). As in the case of the males, distribution of *D*. *melanogaster* females perched on the walls of the Petri dishes with conspecific odorants were significantly different from those obtained in Petri dishes with *D*. *simulans* odorants (Fig 8A and 8D; see below: Statistical analysis of resting place selection and S1 Table). Similar results were obtained for the distribution of females perched on the ceiling (Fig 8B and 8F) and floor (Fig 8C and 8G) of Petri dishes with conspecific odorants and Petri dishes with *D*. *simulans* odorants (see below: Statistical analysis of resting place selection and S1 Table).

#### *D*. *simulans* males

Fig 9A–9G shows perch preferences of non-virgin and virgin *D*. *simulans* males in Petri dishes with conspecific and *D*. *melanogaster* odorants. Most *D*. *simulans* males perched on the walls of Petri dishes (Fig 9A and 9D). Distribution patterns of non-virgin and virgin males of *D*. *simulans* detected on the wall, ceiling and floor of Petri dishes with conspecific odorants were significantly different than in Petri dishes with *D*. *melanogaster* odorants (Fig 9A–9G; see below: Statistical analysis of perch site selection and S1 Table).

#### *D*. *simulans* females

In agreement with the above results, perch preferences of non-virgin and virgin *D*. *simulans* females on the wall, ceiling and floor in Petri dishes with conspecific odorants differed from those in Petri dishes with *D*. *melanogaster* odorants (Fig 10A–10G; see below: Statistical analysis of perch site selection and S1 Table). Taken together, the results of perch preferences of non-virgin and virgin adult *D*. *melanogaster* and *D*. *simulans* indicate that odor cues emanating from the sexes of the two species play an important role in the selection of places to perch and rest.

### Statistical analysis of perch site selection

Perch selection data were analyzed with a multinomial logistic regression (see Materials and methods and S1 Table). Because the floor of Petri dishes was scarcely used by the flies to perch, we used it as a Base comparator [35]. The statistical analysis indicates that odor x sex x sexual experience x species interaction explains intra-and inter-specific differences in perching preferences in Petri dishes: i) preference for wall of Petri dishes yielded an odds ration value = 0.04, confidence interval [0.01–0.19], *P*-value > |z|< 0.05 = 0.001; ii) ceiling preference produced an odds ratio value = 0.16, confidence interval [0.03–0.92], *P*-value > |z|< 0.05 = 0.04. We conclude that adult odors of the same and the sibling are important in perch site selection of *D*. *melanogaster* and *D*. *simulans* adult flies.

### Dispersal orientation

#### *D*. *melanogaster* males

**Fig 11:**
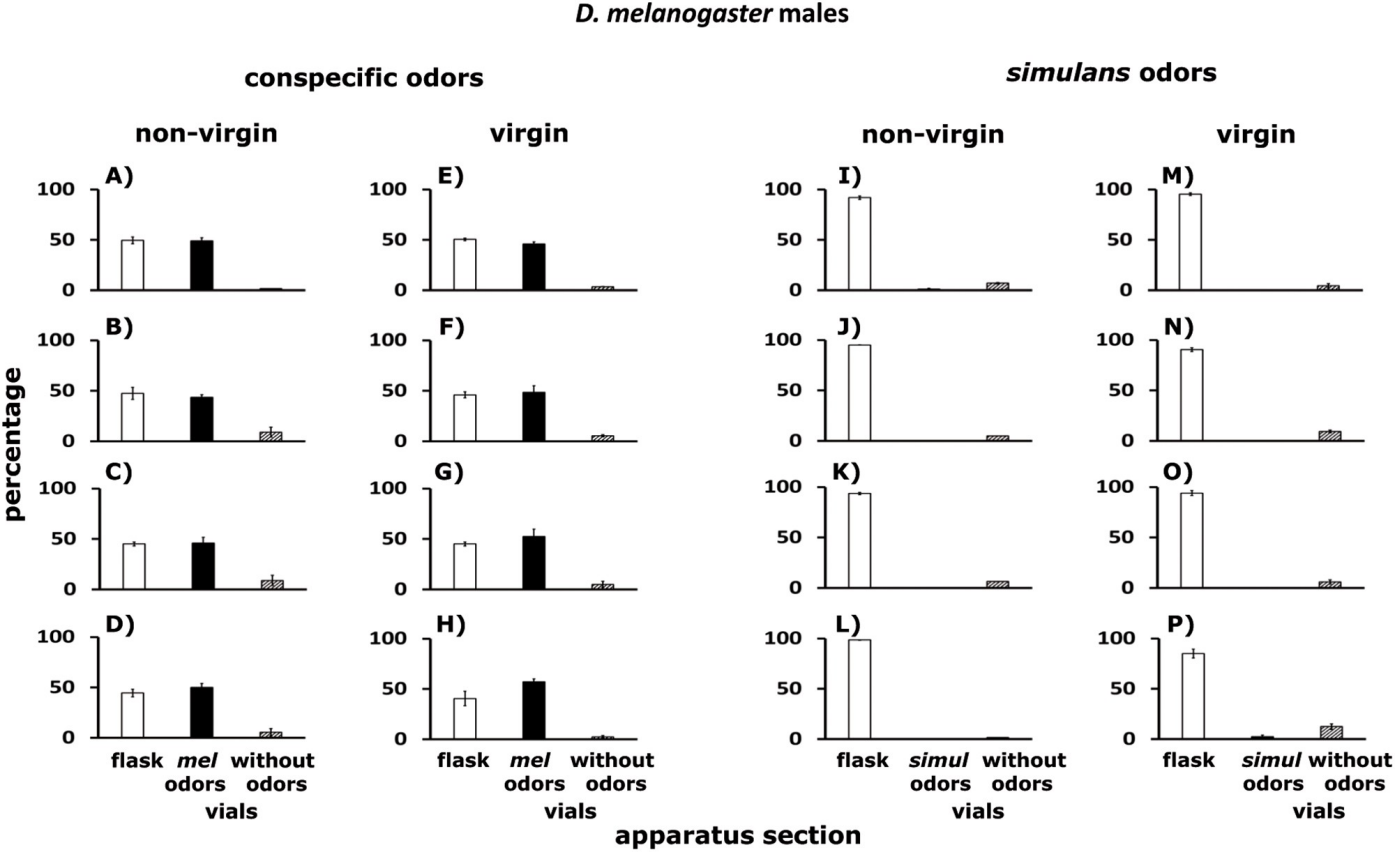
A–P. Dispersal of *D*. *melanogaster* males confronted with odors of conspecifics, and odors of *D*. *simulans*. Males tested were non-virgin (A–D and I–L) and virgin (E–H and M–P). White columns indicate percentages of flies that remained in the flask. Black columns stand for percentages of flies found in the Y-tubes connected to vials with conspecific (or *D*. *simulans*) odorants. Hatched columns show percentages of flies in the Y-tubes connected to empty vials. Conspecific odorants came from: (i) non-virgin males (A, E), (ii) virgin males (B, F), (iii) non-virgin females (C, G), (iv) virgin females (D, H). *D*. *simulans* odorants were emitted by: (i) non-virgin males (I, M), (ii) virgin males (J, N), (iii) non-virgin females (K, O), (iv) virgin females (L, P). See Fig 6.

Fig 11A–11H shows that, stimulated by odorants of conspecific adults, about 50% of non-virgin (A–D) and virgin (E–H) male *D*. *melanogaster* remained in the flask (white columns), another 49% were detected in the Y-tubes connected to vials with conspecific odorants (black columns), while 1.0% of males were in the Y-tubes connected to empty vials (hatched columns). Conspecific odorants came from: (i) non-virgin males (Fig 11A and 11E), (ii) virgin males (Fig 11B and 11F), (iii) non-virgin females (Fig 11C and 11G), (iv) virgin females (Fig 11D and 11H).

Fig 11I–11P shows dispersal of non-virgin (I–L) and virgin (M–P) male *D*. *melanogaster* confronted with odorants of *D*. *simulans*. About 98% of non-virgin and virgin *D*. *melanogaster* remained in the flask (white columns), while 2% were in the Y-tubes connected to empty vials (hatched columns). Odorants of *D*. *simulans* came from: (i) non-virgin males (I, M), (ii) virgin males (J, N), (iii) non-virgin females (K, O), (iv) virgin females (L, P). See below statistical analysis.

#### *D*. *melanogaster* females

**Fig 12:**
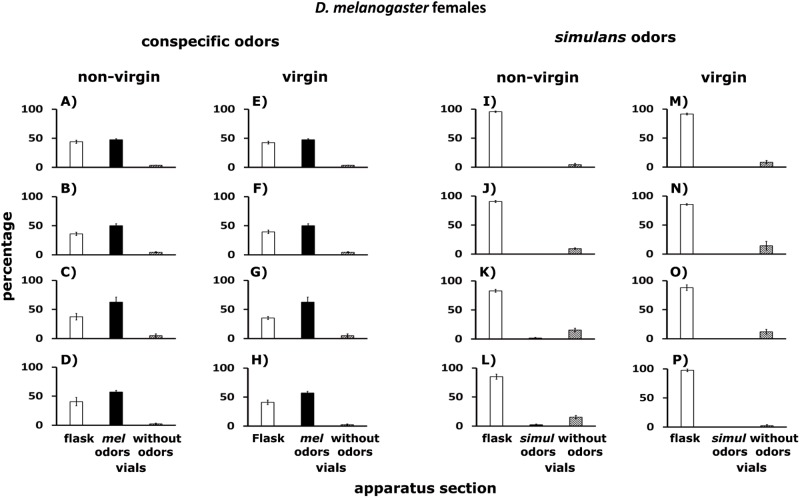
A—P. Dispersal patterns of *D*. *melanogaster* females confronted with odorants of conspecifics, and odorants of *D*. *simulans*. Females tested were non-virgin (A–D and I–L) and virgin (E–H and M–P). White columns indicate percentages of flies that remained in the flask. Black columns stand for percentages of flies found in the Y-tubes connected to vials with conspecific odorants. Hatched columns show percentages of flies in the Y-tubes connected to empty vials. For further details see Fig 11.

49–48% of non-virgin (Fig 12A–12D) and virgin (Fig 12E–12H) female *D*. *melanogaster* remained in the flask (white columns); similar percentages were found in the Y-tubes connected to vials with conspecific odorants (black columns), while 1–2% of the females were observed in the Y-tubes connected to empty vials (hatched columns). Conspecific odorants came from: (i) non-virgin males (A, E), (ii) virgin males (B, F), (iii) non-virgin females (C, G), (iv) virgin females (D, H).

Confronted with odorants of *D*. *simulans* (Fig 12I–12P), about 95% of non-virgin (I—L) and virgin (M—P) female *D*. *melanogaster* remained in the flask (white columns), while 5% were observed in the Y-tubes connected to empty vials (hatched columns). Odorants of *D*. *simulans* came from: (i) non-virgin males (I, M), (ii) virgin males (J, N), (iii) non-virgin females (K, O), (iv) virgin females (L, P). See below statistical analysis.

#### *D*. *simulans* males

**Fig 13:**
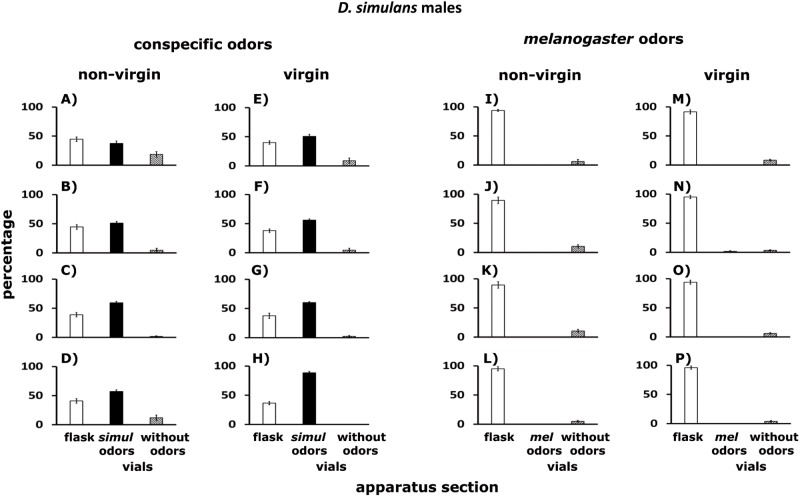
A–P. Dispersal of *D*. *simulans* males confronted with odorants of conspecifics and *D*. *melanogaster*. Males tested were non-virgin (A–D and I–L) and virgin (E–H and M–P). White columns indicate percentages of flies that remained in the flask. Black columns stand for percentages of flies found in the Y-tubes connected to vials with conspecific (or *D*. *simulans*) odors. Hatched columns show percentages of flies in the Y-tubes connected to empty vials. For further details see Fig 11.

Stimulated by odorants of conspecifics of the two sexes, about 47–49% of non-virgin and virgin male *D*. *simulans* remained in the flask (white columns); an additional 50% of such males preferred the Y-tubes connected to vials with odors of conspecifics (black columns), while 1–3% of the males were in the Y-tubes connected to empty vials (hatched columns) (Fig 13A–13H). Conspecific odors came from: (i) non-virgin males (A and E), (ii) virgin males (B and F), (iii) non-virgin females (C and G), (iv) virgin females (D and H).

In response to odorants of *D*. *melanogaster*, about 97% of non-virgin and virgin male *D*. *simulans* remained in the flask (black columns), another 3% were found in the Y-tubes connected to empty vials (hatched columns). Odorants of *D*. *melanogaster* came from: (i) non-virgin males (Fig 13I and 13M), (ii) virgin males (Fig 13J and 13N), (iii) non-virgin females (Fig 13K and 13O), (iv) virgin females (Fig 13L and 13P). See below statistical analysis.

#### *D*. *simulans* females

**Fig 14:**
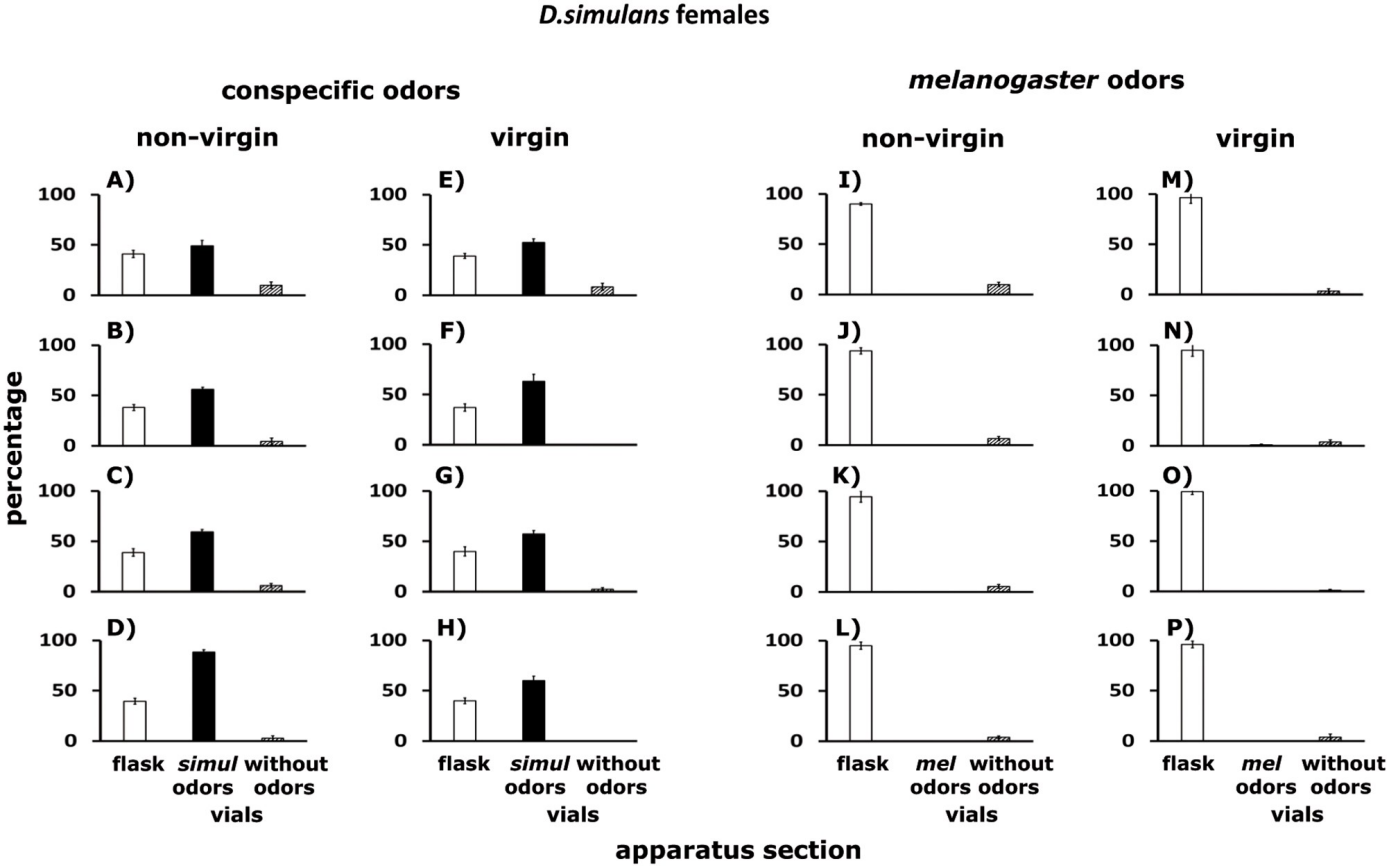
A–P. Dispersal of *D*. *simulans* females confronted with odorants of conspecifics and of *D*. *melanogaster*. Non-virgin (A–D and I–L) and virgin (E–H and M–P) females were tested. White columns indicate percentages of flies that remained in the flask. Black columns stand for percentages of flies found in the Y-tubes connected to vials with conspecific odorants. Hatched columns show percentages of flies in the Y-tubes connected to empty vials. For further details see Fig 11A–11P.

Dispersal patterns of non-virgin (Fig 14A–14D) and virgin female (Fig 14E–14H) *D*. *simulans* confronted with odorants of conspecifics of the two sexes. About 48% of the females remained in the flask (white columns), an additional 50% were in the Y-tubes connected to vials with conspecific odorants (black columns), while 2% were in the Y-tubes connected to empty vials (hatched columns). Conspecific odorants came from. (i) non-virgin males (A and E), (ii) virgin males (B and F), (iii) non-virgin females (C and G), and (iv) virgin females (D and H).

In response to odorants left by *D*. *melanogaster* imagoes, about 98% of non-virgin (Fig 14I–14L) and virgin (Fig 14M–14P) female *D*. *simulans* remained in the flask (white columns) and 2% were in the Y-tubes connected to empty vials (hatched columns). Odorants of *D*. *melanogaster* came from: (i) non-virgin males (I and M), (ii) virgin males (J and N), (iii) non-virgin females (K and O), and (iv) virgin females (L and P). See statistical analysis below.

### Statistical analysis of dispersal

We applied a generalized linear model to evaluate dispersal of *D*. *melanogaster* and *D*. *simulans* in the presence of conspecific odors and adult odors of the sibling. The independent explanatory variables were odor, sex, sexual experience, species, and the corresponding interactions (S2 Table). The odor and species variables exhibit a comparatively reduced width of the confidence intervals for the corresponding estimator coefficients, [1.01–1.16] and [-0.42–-0.04], respectively, suggesting small standard deviations of the estimator coefficients (S2 Table). Additionally, the variables are statistically significant as shown by their respective *P*-values, 0.00001 and 0.019 (S2 Table), suggesting that they explain intra-and inter- specific differences in the dispersal of *D*. *melanogaster* and *D*. *simulans* (S2 Table). The other variables, i.e., sexual experience and odor x species interaction, show larger confidence intervals for the corresponding estimator coefficients and/or non-significant probabilities, suggesting a minor role in explaining intra-and inter-species dispersal differences (S2 Table).

### Results of the two control treatments

In the absence of vials with conspecific odorants and odorants of the sibling species, approximately 95% of non-virgin and virgin male and female *D*. *melanogaster* and *D*. *simulans* stayed in the flask, while 5% of the flies were randomly distributed in the Y-tubes.

In the treatment in which non-virgin and virgin flies of the two species had to move toward vials with conspecific odorants or odorants of the other species, approximately 70% of the flies of each species remained in the flask, and 30% were found in the Y-tubes connected to vials impregnated with conspecific odorants. The results of the two control treatments agree with the findings summarized in Figs 11A–11P to 14A–14P.

## Discussion

We addressed current limitations in the analysis of *Drosophila* adult behavior in the wild by examining populations of *D*. *melanogaster* and *D*. *simulans* in three ecologically different fruit orchards. In each of these locations, adults of the two species showed a preponderance of certain specific behaviors. Our findings suggest that the home range of a number of *Drosophila* species includes decaying fruits and, principally, a variety of microhabitats that surround the fruits. Such microenvironments differ in physical and chemical features such as temperature, illumination conditions, degree of humidity and pH, which influence perch preferences, fly cluster formation, courtship and mating, egg-laying site selection and use of space by *D*. *melanogaster* and *D*. *simulans*. To our knowledge, this is one of the first large examinations of the association between changing, complex environments and a large set of *Drosophila* adult behaviors. Therefore, our results have extensive implications for the evolution of species in the genus *Drosophila*.

### *Drosophila* adult routines in the orchards

An important long-range result of our work is that strong selective forces seem to be operating on behavioral traits to adapt adult *Drosophila* to the ecological conditions found in different orchards. From 9:00 am to 6:00 pm in the grape orchard, *D*. *melanogaster* and *D*. *simulans* adults formed conspecific groups protected from the sun by the canopy (Table 1, Figs 2 and 3). Meanwhile, in the prickly pear orchard (Fig 4), despite temperatures >45 °C adults of the two species moved around, feeding, courting and mating. The two types of orchards are separated by approximately 2 km, a distance typically covered by adult *Drosophila* [36]. In the laboratory, the strains of *D*. *melanogaster* and *D*. *simulans* formed with imagoes collected in prickly pear orchard may live until temperatures of 44 °C (adults) and 52 °C (larvae); the strains formed with individuals from grape orchard remain alive until 38 °C (adults) and 42 °C (larvae). Populations of *D*. *melanogaster* and *D*. *simulans* faced the loss of water due to the high temperatures in the prickly pear orchard (Fig 4); this is opposite to the grape orchard, in which temperatures did not exceed 25 °C (Fig 1). Korol et al. [37] and Michalak et al. [38] have reported ecological and genetic differentiation of natural populations of *D*. *melanogaster* and *D*. *simulans* separated by small distances. The authors think that such differences reflect genetic adaptation to local microclimates [37, 38].

We conjecture that populations of the sibling species that live in the prickly pear orchard could have types of cuticle lipids and hydrocarbons that protect from water loss caused by high temperatures; in *Drosophila*, these molecules have the dual function of preventing loss of water and stimulating courtship and mating [39]. We speculate that such molecules differ from those that coat conspecifics living in the grape orchard where summer temperatures are clearly the lowest. If individuals settled in the prickly pear orchard have particular types of lipids and hydrocarbons coating their bodies, they could also exhibit differences in sexual behavior compared with conspecifics inhabiting the grape orchard. Future work should test this hypothesis. Examples of direct links between specific behaviors and habitats used by *Drosophila* species are rare. This information is important to identify evolutionary pressures on the genes shaping the cuticular chemistry and nervous system of larvae and adults that lead to divergence between populations and species of *Drosophila*.

### Courtship and mating in the wild

Our observations in the wild provide insight into behaviors that biologists have long studied in laboratory conditions, such as courtship and mating [40, 41]. We detected a clear association between the type of courtship and mating and the type of microhabitat. On the dry leaves of grape plants, a male courted and mated with a female in the presence of 3–4 males. The group used the discretionary space available to move around. In the same orchard on unfermented grains of grape that had fallen on the ground, a male courted and mated with a female; the partners used the reduced space allotted by the grain of the grape and participation of other males was not observed. We conjectured that these two types of reproductive behavior could correspond, separately, to *D*. *melanogaster* and *D*. *simulans*. That is, in the wild *D*. *melanogaster* adults could mate in a microhabitat different from that used by *D*. *simulans* adults. Future work should examine this hypothesis. We also conjecture that courting behavior with cooperation of several males could be a result of evolution of *Drosophila* species that coexist in the same sites. The participation of several conspecific males could strengthen the role of pheromones and visual and acoustic signals in the mate recognition system of the species.

Notably, we found a clear space allocation for courtship and mating for three *Drosophila* species in the prickly pear orchard: (i) *D*. *melanogaster* adults courted and mated principally on the fruits of the plant, (ii) both sexes of *D*. *simulans* were detected principally on the cladodes of prickly pear fallen on the ground, and (iii) *D*. *buzzati* males and females met principally on cladodes of the plant. These results and those from the grape orchard provide insights into the evolutionary trajectories of *Drosophila* species.

### Scanning fruits

In grape orchard, females of *D*. *melanogaster* and *D*. *simulans* carefully examined grape grains. They arrived at the fruit in groups of 2–3, but only one female scanned the fruit for approximately 10 min. Interestingly, *D*. *melanogaster* or *D*. *simulans* adults emerged from 99% of the grape grains scanned that contained eggs or larvae, suggesting that the females scan the fruit in search of the presence of pre-adults of the same or the other species. These findings are compatible with the results of del Solar and Godoy-Herrera [42]. These authors found that female *D*. *melanogaster* and *Drosophila funebris* detected the presence of eggs and larvae and rejected laying their eggs in places that had pre-adults of the other species. On the other hand, Bartelt et al. [25] reported that male *D*. *melanogaster* leave pentane extracts on food surfaces that increase their attractiveness to males and females of the same species. Those materials could also induce aversive responses in females of other *Drosophila* species and prevent them from depositing eggs.

In contrast to results in the grape orchard, adult *D*. *melanogaster* and *D*. *simulans* in the prickly pear orchard mostly refrained from scanning fruits and cladodes. We speculate that the Argentinian ants in the prickly pear orchard, which can capture *Drosophila* larvae and adults, may have caused the absence of this behavior. We conjecture that the slow and laborious process of scanning fruit is an obstacle to a fast escape from the ants. Our observations in the prickly orchard suggest that flies detect and react in seconds to escape from a troop of aggressive ants. In the grape orchard, the flies scanned fruits because there are few or no ants (unpublished data). We conclude that the ecological differences between grape and prickly pear orchards provide circumstances for the evolution of discrete behaviors that differentiate natural isolates of *D*. *melanogaster* and *D*. *simulans*.

### Larva-adult behavioral interactions in the wild

Feeding, egg-laying site selection, courtship and mating are complex behaviors that require the entire sensory system [40]. Given this, how does adult *Drosophila* engaged in those activities detect and escape from the ants? The ants arrive in droves at the fruits and cladodes, making the substrate tremble. *Drosophila* larvae seem to detect such vibrations and react by escaping toward the liquefied parts of decaying fruits (or cladodes), digging into and disappearing in the substrate. We speculate that this behavior of *Drosophila* larvae could alert adults to the arrival of the ants. Larva—adult behavioral interactions in *Drosophila* have received very little attention from neurobiologists, geneticists and evolutionists.

### *Drosophila* cluster formation in the wild

The groups formed by adult *D*. *melanogaster* and *D*. *simulans* in the grape orchard (Figs 2, 3 and 5) have the following features: (i) the clusters of one species are physically separated from those formed by the other species (Table 1), (ii) in Petri dishes, the adults of each sibling species spontaneously distribute into groups (Fig 5), (iii) odorants emitted by virgin and non- virgin adult *D*. *melanogaster* and *D*. *simulans* modify the perch preferences of both species (Figs 7–10), (iv) virgin and non—virgin adults orient their movements toward conspecific odorants, avoiding odorants left by individuals of the other species (Figs 11–14). We conclude that clusters observed in the grape orchard seem principally to be a result of response to species-specific odorants that attract conspecifics and repel individuals of the other species (see also Figs 2, 3 and 5). Because non-virgin and virgin flies of both sexes produce odorants (Figs 7–10), we infer that loss of virginity does not affect the production and processing of such signals. These findings contrast with those of sex pheromones, whose production in *Drosophila* depends on the physiological virgin/non-virgin conditions of the flies [43]. Bartelt et al. [25] and Shaner et al. [26] found that the sibling species produce pheromones that induce behaviors comparable to those reported here.

Another important feature of the clusters observed in the wild is the exchange of conspecifics. That is, in the grape orchard, individuals that participate as escorts in courtship activities return to the initial cluster or join with nearby groups of conspecifics. Thus, aggregations observed in the wild seem to be plastic and dynamic, and individuals of a group may mate with flies of other clusters. These features could contribute to decrease inbreeding in wild populations of *D*. *melanogaster* and *D*. *simulans*.

Each vial in which we deposited lies collected in the grape orchard contained either *D*. *melanogaster* or *D*. *simulans*; interspecific hybrids were not found (Table 1), suggesting that mixed groups containing individuals of the two species do not form or are rare in the wild. Our findings on dispersal agree with those observations (Figs 11–14). We conclude that clusters observed in the orchard should be considered a type of pre-zygotic mechanism of reproductive isolation. Nevertheless, this inference is not totally valid. In the apple and prickly pear orchards, we did not check for the presence of interspecific hybrids. Future work should consider the possibility of hybridization between the sibling species in apple and prickly pear orchards. Hybridization between *D*. *melanogaster* and *D*. *simulans* is rare but occurs in the wild because the strength of sexual isolation between populations of the two species varies [32].

### Concluding remarks

Our work in the wild and our experiments in the laboratory have refined the behavioral components of receptivity and rejection in species of *Drosophila* beyond mere copulation acceptance. Our findings show that adults of two non-social species exhibit complex, plastic and changing behaviors with conspecifics and aliens of both sexes in the wild. The behavioral interactions aid in avoiding inter-specific hybridizations and decrease possibilities of intra-and interspecies competition for space and food [8,16]. Therefore, our studies provide an ecological and evolutionary context for the identification of neurons and neuronal circuits that regulate behaviors such as perch preferences, feeding, escape from predators and acceptance and rejection of conspecifics and alien individuals. Whereas we studied the behavior in the wild of *D*. *melanogaster* and *D*. *simulans* in summer and autumn, we know almost nothing about the behavioral adaptations of the adults in winter, when the size of Chilean *Drosophila* populations decreases [28]. How much do the behaviors studied in this investigation extend to other species in the genus *Drosophila*? Interestingly, Godoy-Herrera and Fenner [44] found that non-virgin males and females of South American endemic *Drosophila pavani* responded to odorants emitted by conspecifics. On the other hand, Del Pino et al. [45] found that adults of the Oregon R-c laboratory stock of *D*. *melanogaster* and adults of a wild type strain of *D*. *simulans* collected in Chile, 50 km southwest of Til-Til, exhibited behaviors similar to those reported here. These findings suggest that the routines described in this study are part of the behavioral repertoire of a number of cosmopolitan and endemic species of *Drosophila*.

Finally, our findings also provided information on how the response of adult *Drosophila* to conspecifics and aliens influences community assembly and patterns of biodiversity within and across different orchards, and through different biogeographic regions. This key but understudied problem is linked with the ecological and evolutionary processes that have given rise to differences between species in the genus *Drosophila*.

## Supporting information

S1 Fig Diagram depicting the essays shown in Fig 6. The species were *D*. *melanogaster* and *D*. *simulans*. In one treament, the sexes of each species chose between Y-tubes connected to vials with non-virgin (or virgin) male (or female) conspecific odors and empty vials. In other treatment, flies of each species opted between vials with odors left by non-virgin (or virgin) adults of the sibling and empty vials (see Materials and methods and Figs 6 and 11–14).
(DOC)

S1 Table Multinomial logistic analysis of perch preferences of males and females of *D*. *melanogaster* and *D*. *simulans*. The flies were deposited within Petri dishes with conspecific odors and with odors of the other sibling species. Non-virgin and virgin males and females of the two species were tested. The flies could perch on the wall, ceiling and floor of Petri dishes. Details in Materials and methods.
(DOCX)

S2 Table Statistical analysis of disperal of non-virgin and virgin males and females of *D*. *melanogaster* and *D*. *simulans*. Dependent variables were number of flies in the flask, and in the Y-tubes. Data were analyzed by applying a generalized linear model based on binomial distribution. Details in Materials and methods.
(DOCX)
