# Supplementary material for: The behavior of adult Drosophila in the wild
Source: PLoS One. 2018 Dec 31;13(12):e0209917. doi: 10.1371/journal.pone.0209917 (PMC6312304; doi:10.1371/journal.pone.0209917)
Supplement: S1 Fig — The species were D. melanogaster and D. simulans. In one treament, the sexes of each species chose between Y-tubes connected to vials with non-virgin (or virgin) male (or female) conspecific odors and empty vials. In other treatment, flies of each species opted between vials with odors left by non-virgin (or virgin) adults of the sibling and empty vials (see Materials and methods and Figs 6 and 11–14). (DOC) [file pone.0209917.s001.doc]

**S1 Fig. Diagram depicting the essays shown in Fig 6**. The species were *D. melanogaster* and *D. simulans*. In one treament, the sexes of each species chose between Y-tubes connected to vials with non-virgin (or virgin) male (or female) conspecific odors and empty vials. In other treatment, flies of each species opted between vials with odors left by non-virgin (or virgin) adults of the sibling and empty vials (see Materials and methods and Figs 6, 11 - 14).

**RESPONSE OF NON-VIRGIN (VIRGIN) MALES AND FEMALES OF *D. melanogaster* AND *D. simulans* TO ODORS OF CONSPECIFICS AND ADULTS OF THE SIBLING**

***D.melanogaster***

***D. simulans***

**DISTRIBUTION OF NON-VIRGIN (VIRGIN) MALES (FEMALES) IN THE Y-TUBES CONNECTED TO**

**VIALS WITH ODORS LEFT BY NON-VIRGIN (OR VIRGIN) CONSPECIFICS AND EMPTY VIALS**

**VIALS WITH ODORS LEFT BY NON-VIRGIN (OR VIRGIN) *D. simulans* ADULTS AND EMPTY VIALS**

**DISTRIBUTION OF NON-VIRGIN (VIRGIN) MALES (FEMALES) IN THE Y-TUBES CONNECTED TO**

**VIALS WITH ODORS LEFT BY NON-VIRGIN (OR VIRGIN) CONSPECIFICS AND EMPTY VIALS**

**VIALS WITH ODORS LEFT BY NON- VIRGIN (OR VIRGIN) *D.* *melanogaster* ADULTS AND EMPTY VIALS**
