# Supplementary material for: The behavior of adult Drosophila in the wild
Source: PLoS One. 2018 Dec 31;13(12):e0209917. doi: 10.1371/journal.pone.0209917 (PMC6312304; doi:10.1371/journal.pone.0209917)
Supplement: S1 Table — The flies were deposited within Petri dishes with conspecific odors and with odors of the other sibling species. Non-virgin and virgin males and females of the two species were tested. The flies could perch on the wall, ceiling and floor of Petri dishes. Details in Materials and methods. (DOCX) [file pone.0209917.s002.docx]

**S1 Table.** Multinomial logistic analysis of perch preferences of males and females of *D.* *melanogaster* and *D. simulans*. The flies were deposited within Petri dishes with conspecific odors and with odors of the other sibling species. Non-virgin and virgin males and females of the two species were tested. The flies could perch on the wall, ceiling and floor of Petri dishes. Details in Materials and methods.

| **Petri dish sections and  independent variables** | **Odds ration** | **[95% Odds ratio confidence interval]** | | | **\|z\|** | **P> \|z\|** |
| --- | --- | --- | --- | --- | --- | --- |
| **Wall** |  |  |  |  |  |  |
| **odor** | **78.20** | **[0.10** | **-** | **61643]** | **1.28** | **0.20** |
| **sex** | **130.41** | **[0.16** | **-** | **109496]** | **1.42** | **0.17** |
| **sexual experience** | **225.07** | **[0.21** | **-** | **237344]** | **1.53** | **0.13** |
| **species** | **42.63** | **[0.05** | **-** | **34551]** | **1.10** | **0.27** |
| **odor x sex** | **0.004** | **[0.00** | **-** | **0.23]** | **2.67** | **0.01** |
| **odor x sexual experience** | **0.01** | **[0.00** | **-** | **0.88]** | **2.02** | **0.04** |
| **odor x species** | **0.01** | **[0.00** | **-** | **0.39]** | **2.42** | **0.02** |
| **sex x sexual experience** | **0.00** | **[0.00** | **-** | **0.20]** | **2.73** | **0.01** |
| **sex x species** | **0.02** | **[0.00** | **-** | **1.06]** | **1.93** | **0.05** |
| **sexual experience x species** | **0.01** | **[0.00** | **-** | **0.47]** | **2.32** | **0.02** |
| **odor x sex x sexual**  **experience** | **75.60** | **[6.04** | **-** | **946.16]** | **3.35** | **0.00** |
| **odor x sex x species** | **80.14** | **[6.51** | **-** | **985.94]** | **3.42** | **0.00** |
| **odor x sexual experience x**  **species** | **52.70** | **[4.18** | **-** | **664.72]** | **3.07** | **0.00** |
| **sex x sexual experience x**  **species** | **62.57** | **[4.93** | **-** | **793.30]** | **3.19** | **0.00** |
| **odor x sex x sexual**  **experience x species** | **0.04** | **[0.01** | **-** | **0.19]** | **4.06** | **0.00** |
| **Constant** | **0.65** | **[0.00** | **-** | **54895]** | **0.07** | **0.94** |
|  |  |  |  |  |  |  |
| **Ceiling** |  |  |  |  |  |  |
| **odor** | **0.015** | **[0.00** | **-** | **23.59]** | **1.12** | **0.26** |
| **sex** | **0.273** | **[0.00** | **-** | **434.05]** | **0.35** | **0.73** |
| **sexual experience** | **0.61** | **[0.00** | **-** | **1110.89]** | **0.13** | **0.90** |
| **species** | **0.07** | **[0.00** | **-** | **117.82]** | **0.70** | **0.49** |
| **odor x sex** | **0.70** | **[0.01** | **-** | **60.44]** | **0.16** | **0.87** |
| **odor x sexual experience** | **1.25** | **[0.01** | **-** | **122.66]** | **0.10** | **0.92** |
| **odor x species** | **0.91** | **[0.01** | **-** | **81.72]** | **0.04** | **0.97** |
| **sex x sexual experience** | **0.10** | **[0.00** | **-** | **8.74]** | **1.02** | **0.31** |
| **sex x species** | **0.44** | **[0.00** | **-** | **42.69]** | **0.35** | **0.72** |
| **sexual experience x species** | **0.30** | **[0.00** | **-** | **27.78]** | **0.53** | **0.60** |
| **odor x sex x sexual**  **experience** | **5.30** | **[0.33** | **-** | **85.45]** | **1.18** | **0.24** |
| **odor x sex x species** | **5.40** | **[0.32** | **-** | **89.76]** | **1.17** | **0.24** |
| **odor x sexual experience x**  **species** | **3.80** | **[0.23** | **-** | **62.62]** | **0.93** | **0.35** |
| **sex x sexual experience x**  **species** | **9.35** | **[0.56** | **-** | **156.78]** | **1.55** | **0.12** |
| **odor x sex x sexual**  **experience x species** | **0.16** | **[0.03** | **-** | **0.92]** | **2.05** | **0.04** |
| **Constant** | **16253** | **[0.08** | **-** | **3.43 x 10^9^]** | **1.55** | **0.12** |
| **Floor Base comparator** | | | | | | |
