# Supplementary material for: The behavior of adult Drosophila in the wild
Source: PLoS One. 2018 Dec 31;13(12):e0209917. doi: 10.1371/journal.pone.0209917 (PMC6312304; doi:10.1371/journal.pone.0209917)
Supplement: S2 Table — Dependent variables were number of flies in the flask, and in the Y-tubes. Data were analyzed by applying a generalized linear model based on binomial distribution. Details in Materials and methods. (DOCX) [file pone.0209917.s003.docx]

**S2 Table**. Statistical analysis of disperal of non-virgin and virgin males and females of *D.* *melanogaster* and *D. simulans.* Dependent variables were number of flies in the flask, and in the Y-tubes. Data were analyzed by applying a generalized linear model based on binomial distribution. Details in Materials and methods.

| **Independent variables and interactions** | **Coeficient*** | **[95% Confidence interval]** | | | **\|z\|** | **P> \|z\|** |
| --- | --- | --- | --- | --- | --- | --- |
| **odor** | **1.09** | **[1.01** | **-** | **1.16]** | **29.04** | **0.000** |
| **sex** | **-0.30** | **[-0.54** | **-** | **-0.05]** | **2.36** | **0.018** |
| **sexual experience** | **0.01** | **[-0.24** | **-** | **0.25]** | **0.05** | **0.956** |
| **species** | **-0.23** | **[-0.42** | **-** | **-0.04]** | **2.34** | **0.019** |
| **odor x sex** | **0.09** | **[-0.07** | **-** | **0.24]** | **1.08** | **0.281** |
| **odor x sexual experience** | **-0.04** | **[-0.19** | **-** | **0.11]** | **0.51** | **0.608** |
| **odor x species** | **0.10** | **[-0.01** | **-** | **0.21]** | **1.78** | **0.075** |
| **sex x sexual experience** | **-0.04** | **[-0.42** | **-** | **0.33]** | **0.22** | **0.827** |
| **sex x species** | **0.12** | **[-0.17** | **-** | **0.42]** | **0.82** | **0.412** |
| **sexual experience x species** | **-0.28** | **[-0.59** | **-** | **0.02]** | **1.85** | **0.064** |
| **odor x sex x sexual experience** | **0.08** | **[-0.16** | **-** | **0.31]** | **0.65** | **0.517** |
| **odor x sex x species** | **0.00** | **[-0.19** | **-** | **0.18]** | **0.02** | **0.985** |
| **odor x sexual experience x species** | **0.18** | **[0.01** | **-** | **0.36]** | **2.02** | **0.043** |
| **sex x sexual experience x species** | **0.23** | **[-0.20** | **-** | **0.66]** | **1.04** | **0.297** |
| **odor x sex x sexual experience x species** | **-0.14** | **[-0.41** | **-** | **0.12]** | **1.06** | **0.289** |
| **Constant** | **-2.28** | **[2.40** | **-** | **-2.16]** | **36.88** | **0.000** |
| ***Estimator of corresponding parameter** |  |  |  |  |  |  |
